# The Neurogenic Niche: Interactions Among Vessels, Glia, and Neural Stem Cells

**DOI:** 10.1155/sci/8440764

**Published:** 2026-04-12

**Authors:** Khodakaram Jahanbin

**Affiliations:** ^1^ Department of Immunology, School of Medicine, Ahvaz Jundishapur University of Medical Sciences, Ahvaz, Iran, ajums.ac.ir

**Keywords:** adult neurogenesis, brain plasticity, cerebrovascular circulation, glia, neurogenic niche, neuroinflammation, stem cells

## Abstract

Adult neurogenesis, the generation of new neurons in the adult brain, acts as a fundamental driver of neural plasticity within specialized microenvironments. The integrity of the hippocampal subgranular zone, essential for pattern separation and mood regulation, relies on a functional syncytium formed by the vasculature, glial cells, and neural stem cells (NSCs). This review delineates the architecture of this system, detailing how the vascular pillar provides angiocrine support via vascular endothelial growth factor (VEGF) and brain‐derived neurotrophic factor (BDNF), while the glial pillar—comprising astrocytes and microglia—orchestrates metabolic homeostasis and immune surveillance. The dynamic regulation of this local ecosystem by systemic factors, including physical exercise and the gut–brain axis, is also explored. Furthermore, the breakdown of this alliance is examined as a pathological hub in aging, Alzheimer’s disease (AD), and chronic stress. Crucially, the text addresses the significant translational gap between rodent models and human physiology. The ongoing controversy regarding the persistence of adult human neurogenesis is critically evaluated, attributing conflicting data to methodological variables such as postmortem interval (PMI) and fixation kinetics. Additionally, the risks of maladaptive plasticity, where aberrant neurogenesis contributes to conditions like epilepsy, are discussed. Finally, future directions involving high‐resolution omics and imaging are highlighted, emphasizing that therapeutic strategies must navigate the complex biological risks of neural repair.

## 1. Introduction

The adult brain retains a remarkable capacity for plasticity, partially due to the formation of new neurons in specific neurogenic regions. The subgranular zone of the hippocampal dentate gyrus is the most well‐investigated section, where adult hippocampal neurogenesis (AHN) has been functionally implicated in key cognitive and affective processes. A primary role attributed to AHN is pattern separation, the computational process of differentiating similar memories into distinct representations [1]. Immature adult‐born granule cells enhance this process by regulating the activity of mature granule cells, promoting the remapping of place cells, and improving the precision of memory encoding [2, 3]. Computational models and lesion studies support this framework, demonstrating that the heightened plasticity of young neurons is integral for maintaining memory fidelity and preventing interference [4]. The ablation of neurogenesis impairs the ability to distinguish between closely related experiences, reinforcing the importance of AHN in cognitive flexibility [5]. In addition to cognition, AHN is linked to mood regulation and stress resilience. Chronic stress is a potent negative regulator of AHN, while many antidepressant treatments enhance the production of new neurons [6]. However, this is not a simple causal link, as the mere addition or removal of adult‐born neurons is inadequate to produce antidepressant‐like effects or induce mood disorders [6]. The efficacy of treatments like fluoxetine involves both neurogenesis‐dependent and neurogenesis‐independent mechanisms, which include increasing brain‐derived neurotrophic factor (BDNF), modulating inflammatory pathways, and regulating astrocytic activity [7, 8]. A reciprocal relationship exists between AHN and the hypothalamus–pituitary–adrenal (HPA) axis, where neurogenesis may act as a buffer enhancing stress resilience [9, 10]. The regulation of this process occurs within the neurogenic niche, a specialized microenvironment where a consortium of cells maintains the necessary molecular environment [11]. The foundation comprises neural stem cells (NSCs), a diverse group of radial glia‐like cells that range from a state of quiescence to activation [12]. The behavior of NSCs is regulated by a complex interaction of signals from adjacent cells—astrocytes, microglia, and oligodendrocyte lineage cells—as well as from the vasculature and distal neural circuits [13, 14]. Astrocytes provide trophic support, while microglia modulate neurogenesis through phagocytosis and cytokine release, highlighting an essential glial contribution to niche homeostasis [15]. This architecture is embedded within a unique extracellular matrix (ECM) that provides structural support and biochemical cues to regulate NSC fate [16, 17]. Despite robust evidence from rodent models, the extent of AHN in humans remains controversial. Some studies report that hippocampal neurogenesis continues into the tenth decade of life, with impairments in conditions like Alzheimer’s disease (AD) [18], while others suggest it declines sharply after birth [19]. This divergence is largely attributed to methodological challenges, including postmortem tissue quality and marker sensitivity [19, 20]. Normalizing developmental timelines suggests primate neurogenesis may plateau at very low levels [21], making its resolution critical for translating preclinical findings to human health.

This review will explore the intricate architecture and regulation of the adult neurogenic niche, focusing on the tripartite alliance between the vasculature, glial cells, and NSCs. This review will examine how this local system is modulated by systemic physiological signals and neural activity, how its dysfunction contributes to pathology, and what future paradigms may allow us to harness its potential for brain repair and plasticity. A preprint version of this review has previously been published as: Khodakaram Jahanbin. The Neurogenic Niche: Interactions Among Vessels, Glia, and NSCs [22].

## 2. The Local Niche Architecture: A Multipillar System

**Figure 1:**
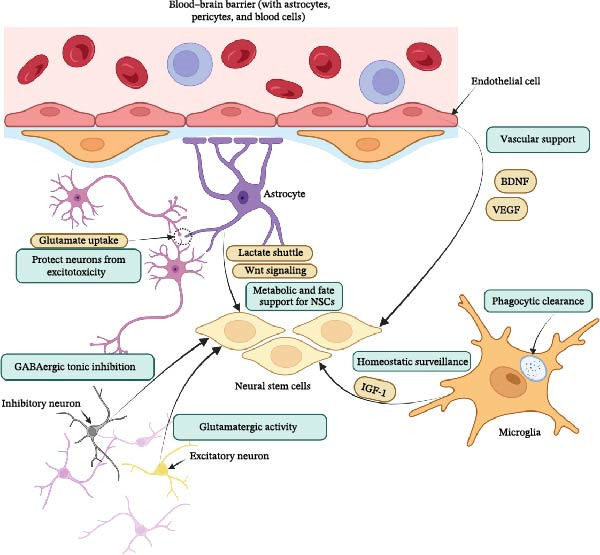
Schematic illustration of the tripartite alliance in the healthy adult neurogenic niche. The central NSCs are supported by interconnected pillars: the vascular system (including endothelial cells, pericytes, and astrocyte end‐feet), providing trophic factors such as BDNF and VEGF for structural and molecular support (note: specialized vascular structures like fractones are specific to the SVZ); the astrocyte pillar, where parenchymal astrocytes facilitate metabolic homeostasis via the lactate shuttle and Wnt signaling, while protecting neurons from excitotoxicity through glutamate uptake; the immune pillar (microglia), enabling homeostatic surveillance through phagocytic clearance of apoptotic cells and secretion of proneurogenic factors like IGF‐1; and top–down neural control, with GABAergic tonic inhibition maintaining NSC quiescence and glutamatergic activity promoting the survival and integration of newborn neurons. This multicellular ecosystem maintains neurogenesis and brain plasticity under homeostatic conditions.

The adult neurogenic niche is a complex, multicellular ecosystem where the fate of NSCs is determined by a sophisticated interplay of local signals. This architecture is built upon a multipillar system comprising the vasculature, distinct glial cell populations, and regulatory neural circuits (Figure 1; Table 1). The vascular system acts as a dynamic regulatory hub, facilitating bidirectional communication essential for homeostasis and integrating systemic signals through direct cell–cell interactions and soluble substances [23, 24]. The astrocytic lineage constitutes a second pillar; while radial glia‐like cells function as the NSCs, distinct populations of parenchymal astrocytes act as indispensable niche cells that provide metabolic support and secrete factors to guide neurogenesis [41, 42]. The immune pillar, composed primarily of microglia, maintains homeostasis through phagocytic clearance and modulates NSC fate via a context‐dependent secretome of pro and antineurogenic factors [15, 88]. Finally, this local machinery is subject to top–down control from neural circuits that use inhibitory, excitatory, and neuromodulatory inputs to precisely gate the stem cell pool and guide the integration of new neurons according to the brain’s computational needs [65, 89]. However, increasingly, this system is understood not merely as separate pillars acting in parallel but as a functional syncytium bound by obligate molecular cross‐talk, discussed in detail in Section 2.4.

**Table 1:** Key components and regulation of the adult neurogenic niche.

Pillar/component	Key structural elements	Primary functions and mechanisms	Key molecular mediators	Role in pathological states
Vascular pillar	Neurovascular unit (endothelial cells, pericytes, astrocytes); high vascular density; basement membrane; fractones (in SVZ).	– Integrates systemic signals with local cues [23, 24]. – Guides NSC fate and differentiation [25, 26]. – Maintains BBB integrity [27, 28].	– VEGF [29, 30] – BDNF [31] – Integrins [32, 33] – TGF and beta [27, 28]	– Aging: vascular senescence, BBB breakdown, reduced perfusion [34, 35] – AD: cerebral amyloid angiopathy, impaired Aβ clearance [36–38] – PD: neurovascular decoupling [36, 39, 40]
Glial pillar: astrocytes	Radial glia‐like cells (serve as NSCs); astrocytic end‐feet form the gliovascular unit.	– Serve as a source of NSCs [41, 42] – Provide metabolic support (lactate shuttle) [43, 44] – Maintain homeostasis (glutamate uptake) [45, 46] – Secrete factors to guide NSC fate and neuronal integration [41, 42, 47, 48]	– Wnt proteins [47] – Ephrin‐B2 [48] – Thrombospondins [49] – IL‐6 (inflammation) [50]	– Injury/disease: reactive astrogliosis forms a glial scar that can be both protective and inhibitory [51] – Aging: senescent astrocytes can become neurotoxic [52]
Glial pillar: microglia (immune)	Resident immune cells of the CNS; exist in homeostatic and reactive states.	– Maintain homeostasis via synaptic pruning [53] – Phagocytic clearance of apoptotic cells, creating a negative feedback loop on neurogenesis [15, 54]	– Proneurogenic: IGF‐1 [55–57], TNFR2 signaling [58] – Antineurogenic: IL‐1&beta [59];, TNF‐α (via TNFR1) [58]	– Aging: inflammaging and transition to a primed, pro‐inflammatory state [60, 61] – Stress/Depression: Drive neuroinflammation [62] – AD: chronic activation creates a hostile cytokine storm [63, 64]
Top–down control: neural circuits	Local inhibitory interneurons (e.g., Parvalbumin+); long‐range GABAergic and cholinergic projections; excitatory glutamatergic inputs	– Inhibitory gating: tonic GABAergic input maintains NSC quiescence [65, 66]. – Activity‐dependent integration: glutamatergic input promotes survival of active new neurons (use it or lose it) [67]	– GABA – Glutamate (NMDA receptors) [68, 69] – α7‐nAChRs [70]	– Epilepsy: seizures lead to aberrant neurogenesis, where new neurons become pro‐epileptogenic [71] – Disruption of inhibitory tone can lead to NSC pool depletion [65]
Systemic and environmental regulation	Gut microbiota; skeletal muscle; peripheral immune system. Influenced by diet, sleep, and toxins	– Exercise: enhances cerebral blood flow and trophic factor release [72, 73] – Gut–brain axis: microbial metabolites modulate microglial function [74, 75]	– Exerkines: irisin, cathepsin B [76–79] – Microbial metabolites: SCFAs (e.g., butyrate) [80, 81]	– Dysbiosis/leaky gut: can lead to systemic inflammation and BBB compromise [82–84] – Chronic stress: elevates glucocorticoids, suppressing neurogenesis [85]. – Toxins: drive neuroinflammation and oxidative stress [86, 87]

*Note:* This table summarizes the primary pillars that constitute the neurogenic niche, including their key structural elements, primary functions, critical molecular mediators, and their roles in various pathological states as discussed in this review.

### 2.1. The Vascular–Neural Crosstalk

The vascular system within neurogenic niches acts as a dynamic regulatory pillar, extending beyond its canonical role of providing oxygen and nutrients. Neurogenic regions are characterized by high vascular density, where neural stem/progenitor cells are organized in close association with blood vessels [90, 91]. This proximity facilitates bidirectional communication essential for nervous system homeostasis and repair, with shared molecular pathways coordinating the synchronic development of both systems [24, 92]. Consequently, the vascular compartment serves as a hub, integrating systemic signals with local cues to influence NSC fate through both soluble blood‐borne factors and direct cell–cell interactions [23]. Disruptions to this neurovascular coupling are implicated in pathologies from cognitive decline to neurodegenerative diseases [24].

#### 2.1.1. Structural Components of the Vascular Niche

The functional core of the vascular niche is the neurovascular unit, a multicellular interface of endothelial cells, pericytes, astrocytes, and neurons that maintains brain homeostasis [27]. Endothelial cells directly influence NSC fate, guiding their differentiation toward neuronal or astrocytic lineages [25, 26]. Pericytes are fundamental for stabilizing the niche (as illustrated in Figure 1, vascular support pillar), where their interactions with endothelial cells regulate angiogenesis, maintain blood–brain barrier (BBB) integrity, and control capillary blood flow through pathways, including PDGF, vascular endothelial growth factor (VEGF), and TGF and beta [27, 28]. Pericyte dysfunction leads to BBB breakdown and is associated with various neurological disorders [93, 94]. A key structural feature is the basement membrane, a specialized ECM that, specifically in the subventricular zone (SVZ), forms unique structures known as fractones [95]. These laminin‐rich bulbs originate from ependymal cells lining the ventricle and directly contact NSCs, acting as reservoirs that concentrate and present growth factors like FGF‐2 to regulate their proliferation [17, 96–98]. While the hippocampal (SGZ) vasculature lacks these ventricular contacts, it relies on a dense capillary network where NSCs interact directly with the endothelial basement membrane.

#### 2.1.2. Molecular Mediators of Neurovascular Crosstalk

Bidirectional communication is orchestrated by a suite of signaling molecules.

##### 2.1.2.1. VEGF

VEGF signaling establishes a powerful positive feedback loop between angiogenesis and neurogenesis [29]. It promotes angiogenesis by binding to VEGF receptor 2 (VEGFR2) on endothelial cells while simultaneously stimulating NSC proliferation and enhancing the survival of newborn neurons, in part by modulating BDNF expression [30]. In pathological contexts like cerebral ischemia, VEGF promotes functional recovery by coordinating both vascular and neural repair [99].

##### 2.1.2.2. BDNF

Cerebral endothelial cells are a major source of BDNF, providing direct trophic support for neuronal survival, maturation, and recruitment through its receptor TrkB [31]. Activation of the BDNF‐TrkB axis triggers downstream ERK and CREB pathways, which are vital for neuronal differentiation and survival, offering broad neuroprotection against various insults [100, 101].

##### 2.1.2.3. Integrin‐Mediated Adhesion

NSCs anchor to the vascular basement membrane through integrin receptors, a process essential for their maintenance. Studies in the SVZ have demonstrated that α6β1 integrin binds to laminin, which is crucial for maintaining NSCs in a quiescent state [32, 33]. Integrin engagement activates intracellular signaling, such as the PI3K/Akt pathway, which supports cell survival and preserves the stem cell pool, and can be influenced by the mechanical properties of the basement membrane [102, 103].

Extracellular vesicle signaling: recent literature highlights a paradigm shift from purely soluble signaling to vesicular transport, where endothelial cells and neural progenitors exchange lipid‐bound nanovesicles to regulate cell fate. Unlike soluble factors, extracellular vesicles (EVs) act as stable carriers for complex molecular cargos, including proteins and microRNAs. Evidence suggests that EC‐derived EVs can modulate neural plasticity and regeneration, though mechanisms vary by tissue type [104]. In the spinal cord, endothelial cells lacking the epigenetic regulator UTX secrete L1CAM‐enriched EVs, which are internalized by NSCs to activate the Akt signaling pathway, thereby promoting neuronal differentiation [105]. Similar EV‐mediated support occurs in the peripheral nervous system, where endothelial cell‐derived exosomes deliver miR‐199a–5p to Schwann cells, stabilizing a repair phenotype via the PI3K/Akt/PTEN axis [106]. Furthermore, the regulation of Wnt/β‐catenin signaling remains a critical aspect of EV‐mediated vascular homeostasis. For example, tumor‐derived endothelial cells utilize miR‐214–3 p and miR‐24–3 p to modulate β‐catenin levels and angiogenesis in an autocrine/paracrine manner [107]. Within the neurogenic lineage, miR‐124 acts as a potent intrinsic regulator, repressing anti‐neurogenic targets such as Sox9 and SCP1 to prioritize neuronal differentiation over gliogenesis [108–110]. While intrinsic expression is the primary driver, therapeutic delivery of miR‐124 via nanoparticles has been shown to boost endogenous repair mechanisms in neurodegenerative models [111]. Finally, hypoxic stress triggers adaptive EV signaling within the niche. Mediated by the HIF‐1α/Rab27a axis, hypoxic NSCs—rather than endothelial cells—increase the secretion of EVs enriched with miR‐210 [112]. These vesicles transfer miR‐210 to neurons, promoting neurite outgrowth and reducing ROS‐induced apoptosis [112], a metabolic modulation distinct from the miR‐210‐mediated ROS increase observed in inflammatory macrophages during atherosclerosis [113].

The decline of cardiovascular health directly compromises these signaling networks, leading to reduced trophic factor secretion, altered vesicular cargo, and a pro‐inflammatory state detrimental to tissue regeneration [114, 115].

#### 2.1.3. Unresolved Questions: The Specificity of Vesicular Transport in the Niche

While the “vesicular hypothesis” posits that EVs serve as sophisticated carriers of complex biological cargo—a view supported by recent findings on pericyte‐mediated neuroprotection and NVU remodeling [116, 117]—the field faces a methodological reckoning regarding the specificity of this signaling in vivo. Although recent studies utilizing genetic editing and click‐chemistry suggest successful endothelial‐to‐NSC targeting [105, 118], transitioning from simplified in vitro models to the dense, lipid‐rich architecture of the living brain reveals significant confounding factors. A primary challenge is the susceptibility of standard tracking technologies to artifactual data. It is now well‐documented that lipophilic dyes (e.g., PKH and DiI) form thermodynamically stable micelles that mimic the size and scatter properties of small EVs, leading to widespread false‐positive staining [119, 120]. In the myelin‐rich environment of the CNS, the high lipid content necessitates rigorous validation to ensure that apparent recipient cells—such as pericytes or NSCs—are not merely retaining dye contaminants or exhibiting altered polarity, a property measurable by solvatochromic probes, rather than true vesicle uptake [121, 122].

Furthermore, genetic reporter systems such as Cre‐LoxP, often regarded as the gold standard, have shown inconsistencies. For example, one study highlighted the complete absence of EV‐mediated Cre mRNA transfer in specific vascular co‐cultures, demonstrating that mRNA packaging is not a universal or guaranteed mechanism [123]. Consequently, a debate persists regarding the fundamental nature of EV targeting in the niche: is it a precise “postal service” or a stochastic broadcast? Proponents of targeted signaling argue for specific address codes, such as unique integrin or tetraspanin profiles [124, 125]. Conversely, biodistribution studies suggest that uptake is often driven by local concentration and clearance rates rather than molecular homing [126, 127].

Most compellingly, emerging high‐resolution data support a “biased stochastic” model [128]. In this view, the unique geometry of the neurovascular unit restricts EV diffusion, creating a high probability of encounter between endothelial donors and perivascular recipients due to spatial confinement rather than strict molecular exclusivity [128, 129]. Whether this involves direct access via apical NSC processes [130] or transcytosis mechanisms mediated by proteoglycans [131] remains a critical frontier requiring advanced in situ analysis.

### 2.2. The Astrocyte Pillar: An Essential Component

The astrocytic lineage constitutes a fundamental pillar of the neurogenic niche (Table 1). While RGL cells function as the NSCs, distinct populations of parenchymal astrocytes act as indispensable niche cells that orchestrate the neurogenic process [41, 132]. These parenchymal astrocytes provide structural support, regulate the local microenvironment, and deliver signals controlling NSC proliferation (Figure 1, astrocyte pillar), fate determination, and neuronal integration [42]. This positions astrocytes at the center of the neuro–immune–vascular axis, where they bridge communication between neurons, immune cells, and the vasculature to maintain CNS homeostasis [133].

#### 2.2.1. Metabolic and Homeostatic Regulation

A primary function of astrocytes is to maintain metabolic and ionic homeostasis. The astrocyte–neuron lactate shuttle is a key mechanism where astrocytes process glucose into lactate, which is then shuttled to neurons as an energy substrate for neurogenesis and synaptic plasticity [43, 44]. Astrocytes are also the primary regulators of extracellular glutamate, expressing transporters that are responsible for 80%–90% of glutamate uptake, thereby protecting neurons from excitotoxicity [45, 46]. They then convert glutamate to glutamine, which is shuttled back to neurons to replenish neurotransmitter pools in the glutamate–glutamine cycle [45].

#### 2.2.2. Structural and Regulatory Roles

Astrocytes form an integral part of the gliovascular unit, where their end‐feet ensheathe the brain’s vasculature, a critical association for inducing and maintaining BBB integrity [134]. They secrete factors like VEGF and TGF‐β that modulate endothelial tight junctions and support vascular health [135]. Their end‐feet are enriched in aquaporin‐4, which regulates water flux and is vital for preventing cerebral edema [136]. Astrocytes also actively shape neural circuits by secreting synaptogenic proteins like thrombospondins [49]. Furthermore, they directly instruct NSC fate through secreted factors like Wnt proteins, which enhance neurogenesis, and juxtacrine signals like ephrin‐B2, which promote neuronal differentiation [42, 47, 48]. Conversely, in response to inflammation, they can secrete factors like IL‐6 that shift NSC fate toward astrogliogenesis [50].

#### 2.2.3. Reactive Astrogliosis: A Context‐Dependent Response

In response to CNS injury, astrocytes undergo reactive astrogliosis, a process with both beneficial and detrimental effects [51]. Protectively, it leads to the formation of a glial scar that isolates damage and supports neuronal survival [137]. However, in chronic pathological conditions, reactive astrocytes can adopt a neurotoxic phenotype that inhibits adaptive plasticity and exacerbates neuronal damage [138].

### 2.3. The Immune–Neural Crosstalk

Microglia, the resident immune cells of the CNS, are dynamic regulators of the neurogenic niche (Table 1). They engage in constant, bidirectional communication with other neural cells to maintain tissue homeostasis and are fundamental to processes like synaptic pruning and the regulation of adult neurogenesis (depicted in Figure 1, immune pillar) [53]. However, while essential for clearing debris, chronic activation can drive neurotoxic inflammation, contributing to neurodegenerative disorders [139, 140]. Current transcriptomic evidence indicates that microglia do not polarize into simple binary states but rather exist on a dynamic functional spectrum, ranging from homeostatic surveillance to various disease‐associated reactive states, which are critical determinants of outcomes within the niche [140, 141].

#### 2.3.1. Phagocytic Regulation of the Stem Cell Pool

A critical homeostatic function of microglia is the phagocytic clearance of apoptotic neural progenitors and newborn neurons, which is essential for maintaining the balance of the stem cell pool [54]. Microglia recognize apoptotic cells via “eat‐me”—molecular cues such as externalized phosphatidylserine that mark dying cells for non‐inflammatory clearance—mediated by receptors including TREM2 and MerTK [141–143]. This engagement not only facilitates engulfment but also actively suppresses pro‐inflammatory signaling [144]. Crucially, this phagocytic act is a key regulatory mechanism; phagocytosing an apoptotic cell triggers a transcriptional program in the microglia, causing it to alter its secretome to limit further neurogenesis, creating a negative feedback loop that ensures stable maintenance of the neurogenic process [15].

#### 2.3.2. The Microglial Secretome: Pro and Antineurogenic Factors

Microglia exert control over NSC fate through the release of soluble factors. Under homeostatic conditions, they release trophic factors like IGF‐1, which promotes NSC proliferation and survival [55–57]. In response to inflammatory stimuli, microglia adopt a reactive, pro‐inflammatory phenotype and release cytokines detrimental to neurogenesis. For instance, the secretome of microglia stimulated by pro‐inflammatory cytokines (e.g., IFN‐γ) has been shown to suppress NSC proliferation [145]. IL‐1β strongly inhibits NSC proliferation via its receptor, IL‐1R1 [59]. The effect of TNF‐α is uniquely pleiotropic; signaling through its TNFR1 receptor is generally associated with neuronal damage and inhibition of proliferation, while signaling through TNFR2 is proneurogenic and required for normal NSC proliferation [58]. The net effect of TNF‐α is thus determined by the balance of signaling through these two pathways.

#### 2.3.3. Sex‐Specific Regulation by Microglia

Microglial function is not uniform between sexes. Adult male and female microglia display distinct transcriptomic profiles, with female microglia often exhibiting a more neuroprotective phenotype [146]. During neonatal development, microglia regulate hippocampal neurogenesis in a sex‐dependent manner, with their depletion impairing the process in males but not females [147]. These differences may be influenced by hormonal factors and can affect brain development, potentially underlying sex‐based disparities in neurological disorders [148].

### 2.4. Interpillar Crosstalk: The Niche as a Network

The reductionist view of the neurogenic niche as a collection of distinct cell types—NSCs supported by a static scaffold of vasculature and glia—is rapidly being supplanted by a systems‐level understanding of the niche as a functionally integrated network. While invertebrate models demonstrate that niche glia can form physical syncytia to manage metabolic and architectural complexity [149], human models reveal that the niche self‐organizes into a highly interconnected ecosystem even without fusion [150]. We now know that the “Three Pillars” are bound together by an obligate, continuous relay of molecular signals, where the output of one cell type serves as the critical input for another [151, 152]. The maintenance of stemness and the successful integration of newborn neurons are governed by the precise integration of these interpillar signals, a homeostatic balance that becomes notably disrupted during aging and immune infiltration [153].

#### 2.4.1. The Microglia–Astrocyte Axis: The “Glial Handshake”

The interaction between microglia and astrocytes represents a primary regulatory axis of the niche, often referred to as the Glial Handshake [53, 154]. This bidirectional communication is a fundamental requirement for synaptic maintenance and the regulation of NSC quiescence [155].

##### 2.4.1.1. Morphogenic Signaling

Central to this crosstalk is the CX3CL1‐CX3CR1‐Wnt signaling cascade. Recent evidence demonstrates that the neuron‐to‐microglia signal CX3CL1 is critical for structural remodeling. Upon activation of the CX3CR1 receptor, microglia secrete Wnt ligands acting paracrinally on neighboring astrocytes. This initiates astrocytic signaling that instructs the retraction of fine perisynaptic processes, effectively “opening up” physical space for neuronal integration [156]. Disruption of this specific ligand–receptor axis, as seen in traumatic injury models, can fundamentally alter the inflammatory trajectory and recovery of the niche [157].

##### 2.4.1.2. Metabolic Signaling

Beyond morphogenesis, this axis governs metabolic energetics. While astrocytes are the primary producers of lactate—a crucial fuel for NSC proliferation and neuronal function [158, 159]—microglia act as key regulators of this supply chain. This relationship is highly sensitive to signaling contexts. Under conditions of immune activation (e.g., LPS stimulation), microglia secrete cytokines such as TNFα, IL‐1β, and IL‐6 via distinct MAPK signaling cascades [160]. These microglial‐derived factors can profoundly alter astrocytic function; for instance, reducing astrocytic inflammatory secretion while modulating their metabolic output [161]. However, the balance is delicate: chronic microglial inflammation has been shown to increase the astrocytic supply of lactate but paradoxically impair its utilization by neurons, leading to metabolic uncoupling [162]. This highlights that while the “metabolic handoff” is vital, its dysregulation by chronic immune signals compromises the bioenergetic fidelity required for neurogenesis.

#### 2.4.2. The Neurovascular Scaffolding: Reciprocal Signaling

The vasculature does not merely supply oxygen but acts as a signaling interface, a concept often paralleled with “angiocrine” signaling in tumorigenesis [163], yet distinct in the healthy niche. This crosstalk relies on a bidirectional molecular dialogue. Research indicates that NSCs actively maintain their own vascular niche; NSCs utilize nitric oxide signaling to stimulate endothelial secretion of VEGF and BDNF, creating a positive feedback loop that preserves vascular tube integrity [164]. Crucially, this dialogue is stabilized by perivascular astrocytes. Contrary to a unidirectional model, astrocytes act as the guardians of the barrier. Astrocyte‐derived Wnt ligands are obligate signals required for the maintenance of the BBB phenotype; in the absence of astrocytic Wnt secretion, endothelial caveolin‐1 expression becomes dysregulated, leading to barrier leakage and niche instability [165]. This communication is heavily reliant on EVs (exosomes), which act as physical vectors for this homeostatic regulation. Unlike simple diffusion, exosomes traffic complex molecular cargo—including active proteins (e.g., HSP70) and specific microRNAs (e.g., miR‐124 and miR‐9)—directly from the endothelium to perivascular astrocytic endfeet [117]. Through this vesicular transport, endothelial signals are capable of modulating astrocytic gene expression and Wnt signaling pathways, thereby reinforcing the structural integrity of the neurovascular unit [166].

#### 2.4.3. The Vascular–Immune Interface

The third pillar of crosstalk occurs at the interface between the vasculature and the immune system, where endothelial cells serve as immunomodulatory gates [167]. This interface is governed by the tight junction protein Claudin‐5, which acts as the primary gatekeeper of paracellular permeability [168, 169]. However, this axis acts as a “double‐edged sword” depending on the signaling context. While controlled permeability allows for surveillance, dysregulated signaling drives niche collapse. Under pathological conditions, such as ischemia, the crosstalk becomes destructive: reactive, pro‐inflammatory microglia release high levels of TNFα, which binds to endothelial TNFR1. Rather than promoting repair, this signal triggers endothelial necroptosis (programmed cell death) and catastrophic BBB disruption [170]. This correlates with a biphasic loss of tight junction proteins (Claudin‐5, Occludin) and the rapid infiltration of immune cells [171]. Thus, the vascular‐immune interface is not a static barrier, but a highly dynamic relay station that can shift from neuroprotection to neurotoxicity based on the integration of microglial and endothelial signals [172].

### 2.5. Top–Down Control by Neural Circuits

The neurogenic niche is dynamically regulated by top–down control from local and long‐range neural circuits, which act as gatekeepers linking the production of new neurons to the computational needs of the broader hippocampal network (Figure 1; Table 1) [65, 66].

#### 2.5.1. Inhibitory Gating and Activity‐Dependent Integration

The maintenance of a quiescent NSC pool is actively enforced by local inhibitory circuits, primarily driven by parvalbumin‐positive interneurons that provide tonic GABAergic input to NSCs [65]. This inhibitory tone holds NSCs in a dormant state; its disruption causes NSCs to exit quiescence, leading to their activation and subsequent depletion [65, 66]. This system is hierarchically controlled by long‐range GABAergic projections from the medial septum [66]. Conversely, excitatory glutamatergic input is essential for the survival and functional integration of newly generated neurons under a “use it or lose it” principle, where the majority of adult‐born neurons undergo apoptosis unless actively recruited into circuits through learning‐dependent activity [67]. This survival is competitively mediated by NMDA‐type glutamate receptors, ensuring that only neurons receiving salient inputs from sources like the entorhinal cortex are retained [68, 69]. Hippocampus‐dependent spatial learning is a primary driver of this selection, coupling neurogenesis directly to cognitive demands [173, 174].

#### 2.5.2. Neuromodulatory Control

Neuromodulatory systems provide another layer of control. Cholinergic inputs from the medial septum play a role in the maturation and integration of adult‐born neurons. Newborn neurons express α7‐containing nicotinic acetylcholine receptors (α7‐nAChRs) and receive direct cholinergic innervation, which is essential for their survival and dendritic development [70]. This relationship is reciprocal: constant adult neurogenesis is necessary to preserve the integrity of the septohippocampal cholinergic circuit throughout life [175].

### 2.6. Key Controversies: The Limits of the M1/M2 Dichotomy and Glial Heterogeneity

While the classification of microglia into binary M1 (neurotoxic) and M2 (neuroprotective) states long served as a dominant heuristic, high‐resolution transcriptomic evidence has rendered this framework largely obsolete in favor of a spectrum of “homeostatic” and “reactive” states [176, 177]. Derived largely from reductionist in vitro studies, the M1/M2 model fails to capture the complexity of microglial biology in the living brain. Indeed, recent data suggest that the binary phenotype is often an artifact of “culture shock”#x2014;transcriptional changes induced purely by removing microglia from their native niche [178]. In vivo, microglia do not toggle between two opposing polarities but exist along a high‐dimensional, dynamic functional spectrum [179]. Single‐cell RNA sequencing (scRNA‐seq) reveals that microglia frequently co‐express markers traditionally assigned to opposing categories (e.g., *Tnf* alongside *Arg1*), creating intermediate phenotypes that the binary model cannot classify [179, 180]. This issue of classification is best exemplified by the discovery of the disease‐associated microglia (DAM) or microglial neurodegenerative phenotype (MGnD) [181, 182]. Unlike cytokine‐polarized states, the DAM/MGnD signature represents a specific reactive phenotype defined by a transcriptional program: the downregulation of homeostatic checkpoints (e.g., *P2ry12* and *Cx3cr1*) and the concurrent upregulation of lipid metabolism and phagocytic pathways (e.g., *Apoe*, *Trem2* and *Lpl*) [183, 184]. Crucially, this state is driven by a *Trem2-ApoE* signaling axis that operates independently of the classical M1/M2 cytokine milieu [185, 186]. Consequently, the field is moving toward a “homeostatic‐reactive” concept that respects the spatiotemporal ontogeny of glial states rather than forcing them into ill‐fitting binary categories.

## 3. Systemic Regulation of the Niche

**Figure 2:**
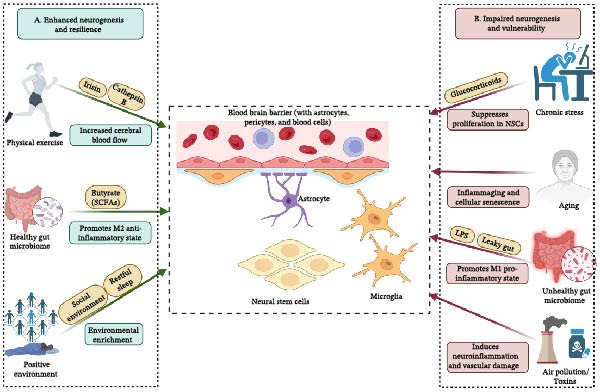
Systemic and environmental modulators of the adult neurogenic niche. (A) Proneurogenic inputs enhancing neurogenesis and resilience, including physical exercise (via irisin and cathepsin B, leading to increased cerebral blood flow), a healthy gut microbiome (via butyrate/SCFAs promoting a homeostatic microglial state), restful sleep, social interaction, and environmental enrichment. (B) Antineurogenic inputs impairing neurogenesis and increasing vulnerability, including chronic stress (via glucocorticoids suppressing NSC proliferation), aging (via inflammaging and cellular senescence), gut dysbiosis (via LPS from leaky gut promoting a reactive microglial state), and environmental toxins (inducing neuroinflammation and vascular damage). The central neurovascular interface (with pericytes and blood cells) integrates these signals, determining the balance between enhanced and impaired neurogenesis.

While the local architecture provides the immediate framework for neurogenesis, the niche does not operate in isolation. Its function is dynamically sculpted by a host of systemic physiological cues and environmental factors that are integrated via the niche vasculature (Figure 2; Table 1). Potent physiological stimuli like physical exercise regulate the niche by enhancing cerebral blood flow and initiating a systemic dialogue through circulating “exerkines”—exercise‐induced signaling molecules such as cytokines, metabolites, and peptides that mediate communication between peripheral organs and the brain—forming a robust body–brain axis [72, 73]. The gut–brain axis represents another critical regulatory network, where the gut microbiota produces metabolites that cross the BBB to influence the niche’s immune landscape [74, 75]. Furthermore, a range of lifestyle and environmental factors, including sleep, environmental enrichment, and exposure to toxins, profoundly modulate the vascular, glial, and immune components of the niche, thereby altering brain plasticity and disease susceptibility [86, 187].

### 3.1. Physiological Cues (Exercise)

Physical exercise is a potent physiological stimulus that regulates the neurogenic niche. A primary mechanism is the enhancement of cerebral blood flow and vascular remodeling [72, 188]. This exercise‐induced hyperemia is mediated by mechanical shear stress on endothelial cells, which activates eNOS signaling to increase nitric oxide bioavailability, a critical vasodilator [189, 190]. This enhanced perfusion is functionally linked to increased expression of VEGF [191]. The VEGF‐C/VEGFR3 signaling axis plays a direct role, as VEGFR3 is expressed on NSCs and its ligand, VEGF‐C, is secreted by endothelial cells to activate quiescent NSCs [192, 193]. In addition to direct vascular changes, exercise initiates a systemic dialogue through circulating factors, or exerkines. The myokine irisin, secreted from muscle tissue, can cross the BBB to stimulate BDNF expression in the hippocampus and has been shown to reduce pro‐inflammatory microglial activation via the TLR4/MyD88 pathway [76, 77]. Irisin is part of a broader orchestra of peripheral factors, including cathepsin B and IGF‐1, that form a robust body–brain axis, triggering cellular changes that enhance neurogenesis and cognitive function (Figure 2A) [78, 79]. The neurogenic response is dependent on the modality and intensity of the activity; sustained high‐intensity aerobic exercise appears most effective, while the effects of resistance exercise are less pronounced and may act through different molecular pathways [194–196].

### 3.2. The Gut–Brain–Niche Axis

The gut–brain axis is a bidirectional communication network where the gut microbiota acts as a central player, producing metabolites that influence neurochemistry and behavior [75, 197]. While clinical data linking dysbiosis to conditions like autism and depression is largely correlational, robust preclinical evidence demonstrates a causal link between the microbiome and neurogenic function [198, 199]. Animal models show that germ‐free mice exhibit impaired neurogenesis in an age‐ and sex‐dependent manner, and transplantation of microbiota from stressed mice into healthy recipients transfers depressive phenotypes via alterations in the endocannabinoid system [198, 200]. This has led to the concept of “psychobiotics”—probiotics capable of influencing mental health [201]. However, translation to humans remains complex. Although a recent meta‐analysis of randomized controlled trials confirms that probiotics may alleviate depressive symptoms, it highlights that no specific strains, dosages, or treatment durations can currently be recommended, indicating a gap between preclinical mechanisms and standardized clinical application [202]. Recent proof‐of‐concept studies in humans have begun to bridge this gap, showing that high‐dose prebiotic fiber can attenuate reward‐related brain activation and shift the microbiome toward short‐chain fatty acid (SCFA) producers, though the precise impact on neurogenesis remains to be visualized in vivo [203].

Mechanistically, the influence of the gut on the niche is mediated by specific humoral and neural pathways. SCFAs—primarily acetate, propionate, and butyrate—are microbial metabolites that cross the BBB to exert epigenetic effects on microglia [74]. Spatial transcriptomic analysis in rodent stroke models reveals that sodium butyrate, a histone deacetylase inhibitor, epigenetically modulates microglia, shifting them from a neurotoxic to a neuroprotective phenotype within the ischemic penumbra [80]. This occurs via the GPR109A/PPAR‐γ/NF‐κB signaling pathway, which suppresses neuroinflammation [81]. The vagus nerve provides a direct anatomical link for this crosstalk [204, 205]. Selective ablation of vagal afferents in rats has been shown to impair hippocampus‐dependent episodic memory and reduce neurotrophic markers, identifying a specific multiorder brainstem–septal pathway connecting the gut to the dorsal hippocampus [206]. Furthermore, constitutive vagal activity is required for the maintenance of BDNF mRNA expression and the survival of complex dendritic arbors in newborn neurons [207].

Gut dysbiosis can initiate a pathological cascade marked by systemic inflammation. Loss of intestinal barrier integrity (“leaky gut”) allows immunogenic substances like bacterial LPS to enter systemic circulation [82]. Sustained exposure to systemic endotoxin triggers the recruitment of peripheral leukocytes—including monocytes and T cells—into the brain parenchyma, altering the inflammatory milieu [82]. In vitro and in vivo models demonstrate that LPS‐activated microglia produce reactive oxygen species that fragment tight junction proteins (e.g., zonula occludens‐1 and claudin‐5) and cause pericyte detachment, thereby compromising the BBB and creating a hostile environment for neurogenesis [83, 84].

### 3.3. Additional Environmental and Lifestyle Factors

Chronic sleep deprivation critically impairs the neurogenic niche by fostering a pro‐inflammatory microenvironment. This is mechanistically linked to the robust activation of microglia, an increase in pro‐inflammatory cytokines like IL‐1β, and a significant decline in BDNF [208]. In contrast, environmental enrichment robustly enhances brain plasticity by upregulating genes associated with neurogenesis and cell survival, enhancing neurotrophin expression, and promoting resilience [187, 209, 210]. Exposure to environmental toxins like air pollutants and heavy metals represents a significant threat (illustrated in Figure 2 for both positive and negative modulators). These toxins disrupt the vascular and glial pillars through neuroinflammation and oxidative stress [86, 87]. Air pollution impairs neurogenesis by stimulating the activation of astrocytes and microglia, while traffic‐related air pollution can cause severe vascular disruption, including a reduction in the tight junction protein ZO‐1 and an increase in microhemorrhages [87, 211, 212]. The interplay between lifestyle, environment, and genetics is also critical in determining risk for neuroinflammatory diseases like multiple sclerosis, where factors such as smoking, EBV infection, and obesity interact with HLA risk genes [213]. Many of these environmental influences are modifiable, offering opportunities for disease prevention.

### 3.4. Key Controversies: Causality vs. Correlation in the Human Gut–Brain Axis

The psychobiotic revolution has established a “causal bedrock” in preclinical models, where specific microbes definitively modulate brain structure and behavior. In rodents, mechanisms are mapped with high fidelity: *Lactobacillus rhamnosus* JB‐1 utilizes the vagus nerve to reduce stress, an effect abolished by vagotomy [214]. Parallel pathways involve metabolic signaling, where SCFAs like butyrate act as histone deacetylase inhibitors, restoring neurogenesis in murine stress models [215] and preventing cytokine‐induced apoptosis in human hippocampal cells [216]. However, human translation remains characterized by a profound gap between these mechanistic insights and clinical reality [217, 218]. While recent meta‐analyses indicate statistically significant benefits for depression (SMD −0.96) and anxiety (SMD −0.59) [219], the data reveal a sharp divergence based on population and methodology [220]. For instance, while *L. Rhamnosus* JB‐1 is potent in mice; it failed to alter stress or cognitive performance in healthy human volunteers [218]. Conversely, in high‐stress clinical populations, such as surgical oncology patients, psychobiotics have demonstrated robust efficacy, reducing depression rates by over 60% [221]. This inconsistency highlights a “precision crisis” [222]. The field struggles with the validation of central mechanisms in humans, as direct evidence of AHN remains methodologically fraught and heavily debated, relying on rare postmortem samples rather than accessible live biomarkers [18, 223].

Finally, while observational studies struggle with the “chicken‐and‐egg” problem of dysbiosis, emerging genetic evidence is beginning to resolve the directionality of these associations. Two‐sample Mendelian Randomization studies have recently identified specific causal bacterial taxa—such as the link between *Prevotellaceae* and autism spectrum disorder—explicitly ruling out reverse causality in these specific pairings [224]. Thus, the challenge is no longer establishing if the gut affects the brain but identifying which strains are effective for which human phenotypes [225].

## 4. Human Translation and Comparative Biology

Translating findings from rodent models to human biology presents significant challenges, rooted in species‐specific biological differences but also in a profound methodological crisis regarding how human tissue is processed and analyzed [226]. The neuro–immune–vascular interface has emerged as a critical nexus for this translation, as dynamic interactions between these systems are pivotal in maintaining homeostasis and responding to stress [227, 228]. The neuro–immune–vascular interface remains a critical nexus for this translation, yet the dynamic interactions observed in mice are difficult to capture in human postmortem samples. To resolve the conflicting reports regarding the persistence of AHN in humans, it is necessary to move beyond descriptive phenomenology and address the specific technical variables—fixation kinetics, postmortem interval (PMI), and autofluorescence—that determine whether neurogenic markers are detected or masked. Furthermore, the integration of multiomic and volumetric imaging approaches offers a path to validate these histological findings mechanistically.

### 4.1. Species‐Specific Architectures of the Neurogenic Niche

Comparative studies reveal profound differences in the organization and temporal dynamics of neurogenic niches across species. While rodent neurogenesis is characterized by rapid maturation cycles lasting weeks, primate neurogenesis is marked by a notably “protracted maturation” period. For example, granule cells in the dentate gyrus of adult macaques take at least 6 months to mature—over six times longer than in rodents [229]. This phenomenon of extended plasticity is particularly evident in the primate amygdala and neocortex. In humans, immature excitatory neurons in the amygdala can persist in a state of deep quiescence for decades, serving as a substrate for persistent plasticity [230]. Similarly, studies in the common marmoset reveal that while hippocampal neurons mature within months, postnatally born neurons in the neocortex remain immature for up to half a year [231]. This extended timeline suggests that the primate brain prioritizes the maintenance of a “neurogenic reserve”—a sustained pool of plastic, immature neurons or stem cells—rather than the high‐throughput proliferation seen in short‐lived mammals [232, 233]. Recent genetic analyses have confirmed human‐specific regulatory patterns, including delayed acquisition of mature neuronal profiles and neoteny, that underlie this prolonged plasticity [234].

### 4.2. The Controversy of Adult Human Hippocampal Neurogenesis

The controversy surrounding human AHN is driven largely by methodological divergence. While some studies report a sharp cessation of neurogenesis in childhood [235], others utilizing optimized protocols demonstrate its persistence into the tenth decade of life [18]. This discrepancy stems from three critical variables that affect the stability and detectability of neurogenic markers like doublecortin (DCX).

#### 4.2.1. Fixation Kinetics and Epitope Masking

The standard practice of immersing whole human hemispheres in formalin leads to prolonged fixation times, often exceeding several weeks. This extended exposure catalyzes the formation of dense methylene bridge cross‐links that sterically hinder antibody binding, effectively “masking” epitopes [236]. Although some antigens are robust, labile markers associated with plasticity are highly sensitive to this cross‐linking. Studies utilizing shorter fixation times (<24 h) or employing aggressive heat‐induced antigen retrieval have successfully unmasked abundant DCX + populations in aged subjects that were invisible in standard preparations [237]. Furthermore, the development of alternative fixatives like glyoxal or glyoxal acid‐free solutions offers promise for better preserving antigenicity in future biobanking [238, 239].

#### 4.2.2. The Age–PMI Interaction

Neurogenic markers are differentially labile. While “housekeeping” proteins like NeuN are stable, cytoskeletal proteins “DCX” and cell cycle markers Ki67 are prone to rapid degradation. Recent experimental work in mice confirms that while fixation time is a dominant variable, the PMI is a critical compounding factor that reduces the visualization of immature neurons, with effects being significantly more severe in aged animals [240]. This age‐by–PMI interaction creates a “floor effect”: in aged brains where baseline neurogenesis is already low, even moderate PMIs delay fixation enough to degrade dendritic arbors to the point where immature neurons resemble small glial cells, leading to false negatives [20]. Rigorous validation, therefore, requires donor selection with minimal PMI to preserve the dendritic integrity essential for morphological identification.

#### 4.2.3. Lipofuscin and Autofluorescence

The accumulation of lipofuscin, an autofluorescent pigment composed of oxidized lipids and misfolded proteins, is a hallmark of the aging human hippocampus [241]. This accumulation introduces a severe signal‐to‐noise problem, as lipofuscin fluorescence overlaps with common imaging channels, leading to the misidentification of glia as neurons (false positives) or the masking of faint signals (false negatives) [242]. To resolve this, modern protocols must employ chemical quenching agents such as Sudan Black B or newer commercial reagents like TrueBlack, which reduce autofluorescence by over 89% without compromising immunolabeling [243]. Alternatively, photobleaching with high‐intensity white light has emerged as a cost‐effective method to eliminate this background signal [244].

### 4.3. Resolving Discrepancies Through Emerging Omics and Imaging

To transcend the limitations of traditional histology, the field is increasingly turning to high‐dimensional modalities that provide mechanistic validation of the neurogenic process [245].

#### 4.3.1. Single‐Nucleus Transcriptomics and Machine Learning

Single‐nucleus RNA sequencing allows for the analysis of biobanked frozen tissue, capable of resolving cellular heterogeneity often lost in bulk analysis [246]. Recent breakthroughs utilizing supervised machine learning have addressed the statistical challenge of detection. A landmark study successfully identified proliferating neural progenitors in the adult human hippocampus that share the transcriptomic signature of developmental neurogenesis, confirming their persistence [247]. Furthermore, these multiomic approaches have defined the molecular landscape of human immature neurons, identifying specific epigenetic barriers (e.g., EZH2 and DOT1L) that orchestrate the protracted maturation phenotype unique to primates [248, 249].

#### 4.3.2. Spatial Transcriptomics

Validating the location of these cells is the final evidentiary step. Spatial transcriptomics technologies (e.g., Visium and MERFISH) enable the mapping of gene expression directly within tissue architecture. While initially deployed to map laminar signatures in the human prefrontal cortex [250], these tools are now essential for distinguishing bona fide immature neurons from inhibitory interneurons or glial cells that may express overlapping markers. This spatial resolution is critical for resolving the “identity crisis” of cells in the adult niche by bypassing tissue dissociation [251, 252].

#### 4.3.3. Volumetric Imaging and Tissue Clearing

Finally, to visualize the complex dendritic arbors of new neurons in 3D, tissue‐clearing protocols optimized for human archival tissue are revolutionizing histological analysis. Techniques such as aDISCO have proven versatile for FFPE blocks, enabling the consistent staining and clearing of samples stored for at least 15 years [253]. Concurrently, methods like SHANEL allow for the cellular mapping of intact, whole human organs [254]. By coupling these clearing techniques with lipophilic tracers, researchers can now visualize dendritic trees and spines in 3D with high resolution, providing morphological proof of neurogenesis without the sampling errors inherent in 2D sectioning [226, 255].

### 4.4. Key Controversies: The Identity Crisis of Adult Human Neurogenesis

The question of whether the adult human brain retains the capacity for neurogenesis remains one of the most polarizing debates in modern neuroscience, characterized by a fundamental epistemological crisis driven by histological methodology [19, 256]. This division is exemplified by diametrically opposed conclusions: while some groups argue that hippocampal neurogenesis extinguishes in childhood [257], others have demonstrated the persistence of thousands of immature neurons into the ninth decade of life [258, 259]. This divergence is largely attributable to the “unholy trinity” of histological artifacts: fixation kinetics, PMI, and lipofuscin autofluorescence [237, 257]. The detection of the gold‐standard marker DCX is mathematically determined by the kinetics of aldehyde fixation; prolonged immersion (weeks to months), typical of standard brain banks, creates dense methylene bridge cross‐links that sterically hinder antibody binding. Indeed, comparative models confirm that fixation time, rather than PMI, is the primary factor in sterilizing the tissue of signal [240]. In contrast, protocols restricting fixation to a 24 h window reveal robust neurogenic populations that are otherwise masked [18, 260]. Furthermore, the accumulation of lipofuscin—an undegradable, autofluorescent lysosomal pigment—in aging neurons creates a severe signal‐to‐noise problem. While traditional quenching (e.g., Sudan Black B) has been standard, recent advances utilize high‐intensity white light photobleaching to eliminate this background without the chemical interference associated with dye‐based quenchers [244, 261]. Beyond these technical barriers lies a biological controversy regarding “dematuration.” Critics argue that even if DCX + cells are detected, they may not represent newly born neurons derived from a stem cell niche, but rather mature granule cells that have reverted to an immature phenotype in response to stress [262]. Resolving this identity crisis requires moving beyond standard immunohistochemistry to integrate spatial transcriptomics. Recent applications of this technology have begun to map the precise molecular phenotype of these cells, distinguishing true neurogenic lineages from ambiguous or demature profiles with unprecedented resolution [263, 264].

## 5. The Breakdown of the Alliance: Pathological Hubs

**Figure 3:**
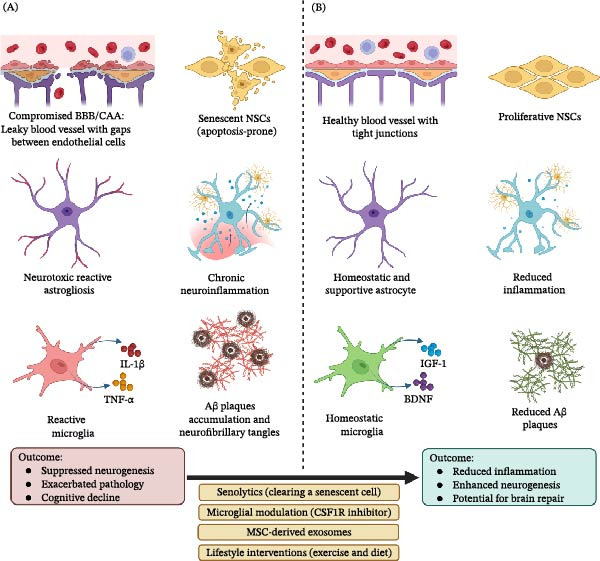
Dysfunction and therapeutic restoration of the neurogenic niche. (A) Pathological state in conditions such as aging, chronic stress, or Alzheimer’s disease, depicting a deteriorated niche with senescent or apoptotic NSCs, compromised BBB integrity and cerebral amyloid angiopathy, neurotoxic reactive astrogliosis, and reactive microglia releasing cytokines (e.g., IL‐1β and TNF‐α), amid hallmarks like Aβ plaques, neurofibrillary tangles, and chronic neuroinflammation. This leads to suppressed neurogenesis, exacerbated pathology, and cognitive decline. (B) Therapeutically restored state following interventions such as senolytics (for senescent cell clearance), microglial modulation (e.g., via CSF1R inhibitors), mesenchymal stem cell (MSC)‐derived exosomes, and lifestyle factors (e.g., exercise and diet), showing rejuvenated NSCs with active proliferation, restored BBB integrity, homeostatic astrocytes, and homeostatic microglia releasing trophic factors (e.g., IGF‐1 and BDNF). This results in reduced inflammation, enhanced neurogenesis, and potential for brain repair.

Given its critical role in brain plasticity, the deterioration of the neurogenic niche is a central feature and common pathological hub across a wide spectrum of neurological disorders (Table 1). In the context of physiological aging, the niche undergoes a significant decline driven by interconnected processes including vascular senescence, chronic low‐grade inflammation (inflammaging), and the accumulation of senescent cells [34, 60]. In conditions like chronic stress and depression, neuroinflammatory processes degrade the niche’s integrity through mechanisms such as HPA axis dysregulation and glucocorticoid‐mediated suppression of NSCs [62]. In neurodegenerative disorders such as AD and Parkinson’s disease (PD), disease‐specific pathologies converge to dismantle the tripartite alliance, transforming a site of plasticity into a driver of disease progression through chronic neuroinflammation, BBB disruption, and aberrant stem cell responses (Figure 3A) [36, 265].

### 5.1. The Aging Niche

Aging precipitates a significant decline in the functional integrity of the neurogenic niche. This deterioration involves a cascade of interconnected processes, including vascular senescence, chronic inflammaging, the accumulation of senescent cells, and epigenetic alterations.

#### 5.1.1. Vascular Deterioration

The age‐related decline of the vascular system is a primary driver of neurogenic failure. Neurovascular aging manifests as impaired oxygen delivery, compromised protein clearance, and BBB disruption, which facilitates the infiltration of peripheral immune cells and exacerbates neuroinflammation [34, 35]. Arterial stiffness and endothelial dysfunction promote a preatherogenic state and are mechanistically linked to impaired angiogenesis and pathological microcirculatory remodeling [266, 267].

#### 5.1.2. Inflammaging and Primed Glia

Aging is characterized by inflammaging. Within the aging brain, microglia transition to a “primed” state—an altered phenotype marked by exaggerated sensitivity to stimuli and amplified inflammatory output—exhibiting a heightened and prolonged inflammatory response to stimuli, resulting in the sustained production of pro‐inflammatory cytokines that contribute to cognitive deficits [60, 61]. Aged microglia also experience defects in homeostatic functions like phagocytosis, creating a self‐perpetuating cycle of neuroinflammation and neurotoxicity [268].

#### 5.1.3. Cellular Senescence

Senescent cells, including astrocytes, microglia, and NSCs, accumulate in the aging brain and adopt a senescence‐associated secretory phenotype (SASP) [52, 269]. While traditionally defined by soluble cytokines, recent evidence identifies EVs (SASP‐EVs) as a critical component of the senescent phenotype [270, 271]. Senescent endothelial cells secrete elevated numbers of EVs containing a pro‐senescent cargo. For instance, senescent human umbilical vein endothelial cells release small EVs enriched with miR‐21–5p and miR‐217, which target DNMT1 and SIRT1 to propagate senescence and inhibit proliferation in neighboring cells [272]. Additionally, proteomic analyses have identified the accumulation of CAP1 in EVs from aged endothelium, which drives senescence and accelerates atheroma plaque formation [273]. When internalized by recipient cells within the niche, these SASP‐EVs can modulate the DNA Damage Response, transmitting senescence via the “bystander effect” [274, 275]. This inflammatory microenvironment poses a dual threat: it compromises the structural integrity of the BBB [276] and disrupts the intercellular configuration of NSCs. Notably, the maintenance of stemness in NSCs relies on tight junction proteins (e.g., ZO‐1 and occludin); their downregulation—often precipitated by niche dysregulation—forces a premature loss of the stem cell pool [277].

#### 5.1.4. Loss of Rejuvenating Signals

The aging niche is further compromised by the depletion of “youthful” vesicular signals [278, 279]. In healthy states, circulating EVs carry regenerative cargos such as α‐Klotho mRNA, which has been shown to restore bioenergetics and regenerative capacity in aged tissues [280]. Furthermore, young EVs maintain metabolic homeostasis by delivering miR‐223–3 p, which directly targets and suppresses the NLRP3 inflammasome [281, 282]. The age‐dependent reduction in these protective cargos, coupled with a failure to stimulate PGC‐1 α‐mediated mitochondrial metabolism [283], prevents the niche from mounting an effective regenerative response [284].

#### 5.1.5. Epigenetic Drift

Aging is accompanied by a dysregulation of transcriptional and chromatin networks, termed “epigenetic drift” [285]. These alterations in the epigenetic landscape of NSCs contribute to their diminished proliferation and increased quiescence, while compromised signaling from aging niche cells accelerates this decline, leading to a collapse of tissue homeostasis [286].

### 5.2. Stress and Depression

Chronic stress is a potent catalyst for neuroinflammatory processes that degrade the niche’s integrity and contribute to major depressive disorder. The neuroimmunoinflammatory stress model posits that depression represents a terminal stage of chronic stress, marked by sustained pro‐inflammatory responses that culminate in neuroinflammation. This involves dysregulation of the HPA axis and heightened inflammation in key mood‐regulating brain regions [62, 287]. A primary consequence is the elevation of glucocorticoids, which potently suppress NSC proliferation by activating GRs on NSCs, triggering cell cycle arrest in the G1/G0 phase through the degradation of cyclin D1 and upregulation of inhibitory genes [85, 288]. Microglia play a pivotal role, as elevated glucocorticoids can prime them toward a pro‐inflammatory state via a GR‐NF‐κB‐NLRP3 inflammasome pathway [288]. Chronic stress also significantly disrupts trophic support, most notably by reducing the expression of BDNF through multiple convergent mechanisms involving glial and vascular dysfunction, including epigenetic suppression of BDNF transcription [287, 289]. Encouragingly, interventions like mindfulness and exercise can modulate these pathways, highlighting the plasticity of the niche as a therapeutic target for stress‐related disorders [290].

### 5.3. AD

In AD, the pathological accumulation of amyloid‐β (Aβ) and hyperphosphorylated tau dismantles the neurogenic niche. The effect of Aβ oligomers on NSCs is complex; high concentrations induce apoptosis, DNA damage, and oxidative stress, yet some studies report context‐dependent neurogenic‐promoting effects [291, 292]. The vascular pillar is severely compromised through cerebral amyloid angiopathy, where Aβ accumulates within cerebral blood vessel walls, impairing Aβ clearance, undermining the neurovascular unit, and leading to BBB compromise and chronic cerebral hypoperfusion [36–38]. Hyperphosphorylated tau also becomes a disruptive force, impeding microtubule dynamics and eliciting neuroinflammatory responses [293, 294]. Finally, chronic neuroinflammation, driven by microglial activation in response to Aβ plaques, creates a hostile “cytokine storm”—an excessive and dysregulated release of pro‐inflammatory cytokines that overwhelms homeostatic signaling—directly suppressing AHN and transforming the niche into one that actively promotes neurodegeneration (Figure 3A) [63, 64].

### 5.4. Broader Pathologies and Comorbidities

Breakdown of the tripartite alliance is a common pathological hub across a spectrum of neurological disorders.

#### 5.4.1. PD

Cognitive deficits arise from neurovascular decoupling, dysregulated microglial activation that creates a vicious cycle with dying neurons, and vascular pathology, including a compromised BBB [39, 40, 265].

#### 5.4.2. Traumatic Brain Injury (TBI)

TBI triggers an acute activation of NSCs, but this regenerative attempt is often thwarted as differentiation is skewed toward an astrocytic fate. The chronic phase is characterized by a glial scar and persistent neuroinflammation that suppresses NSC proliferation and compromises recovery [295].

#### 5.4.3. Epilepsy

Seizures profoundly disrupt hippocampal neurogenesis, transforming it into a pathological driver. The neurogenesis that occurs is aberrant, with newborn granule cells displaying persistent immaturity, migrating to ectopic locations, and contributing to network hyperexcitability, making it proepileptogenic rather than reparative [71].

#### 5.4.4. Vascular and Demyelinating Disorders

In conditions like vascular cognitive impairment and multiple sclerosis, immune‐mediated neurovascular dysfunction is a primary driver. Dysregulated glial activation and infiltration of inflammatory cells promote white matter degeneration, while systemic vascular comorbidities are linked to greater disability, highlighting a bidirectional relationship between peripheral vascular health and central neurodegeneration (Figure 3 for a summary of niche breakdown across pathologies) [296, 297].

### 5.5. Unresolved Questions: Maladaptive Plasticity: When Neurogenesis Goes Wrong

A critical unresolved question is whether the neurogenic response observed in neurological disorders represents a failed reparative attempt (compensatory) or an active driver of pathology (maladaptive) [298, 299]. While the “Neurogenic Reserve Hypothesis” posits that plasticity confers resilience [300, 301], evidence from temporal lobe epilepsy (TLE) and AD suggests that in a corrupted niche, this reserve can paradoxically become a liability. In TLE, the mechanisms of neurogenesis are subverted to support seizure generation [302]. Seizure‐induced proliferation results in Hilar Ectopic Granule Cells—neurons that migrate aberrantly into the hilus. While historically attributed to Reelin loss, recent evidence identifies excitatory GABAergic signaling (driven by upregulated NKCC1) as the force reversing the migration of these newborn cells [303, 304]. These ectopic cells are not bystanders; they exhibit intrinsic hyperexcitability and act as “hub cells” that synchronize the epileptic network [305, 306]. Furthermore, they contribute to “recurrent excitatory loops” via Mossy Fiber Sprouting, bypassing the physiological dentate gate [307, 308]. In AD, the maladaptation is subtler. The “Tau‐mediated aberrant neurogenesis” hypothesis proposes that the high‐plasticity state of newborn neurons makes them uniquely vulnerable to hyperphosphorylation, potentially turning them into “Trojan horses” that act as vectors for spreading Tau pathology [309]. This is compounded by niche corruption, where Tau accumulation in hilar astrocytes further impairs metabolic support and synaptic integration [310]. Additionally, populations of stalled immature neurons—trapped in a state of developmental arrest—contribute to “silent” network hyperexcitability and background noise that degrades memory encoding [311, 312]. This raises a therapeutic dilemma: indiscriminately boosting neurogenesis without correcting the niche could inadvertently accelerate disease by generating pro‐epileptogenic or dystrophic neurons. Thus, the field must resolve whether the primary goal is to enhance neuronal quantity or to first restore the quality of the niche signals—such as bioelectric or metabolic cues—that guide them [313, 314].

## 6. Future Directions and Therapeutic Paradigms

Overcoming the challenges posed by niche dysfunction in aging and disease requires the development and application of innovative technologies and therapeutic strategies that can precisely probe and manipulate this complex microenvironment. Breakthroughs in advanced imaging, such as intravital multiphoton microscopy, combined with sophisticated genetic labeling strategies, are pivotal for visualizing dynamic cellular processes in living systems [315, 316]. The convergence of high‐throughput omics with systems biology is providing an unprecedented, holistic understanding of the niche’s regulatory networks [317]. This deeper understanding is paving the way for novel therapeutic strategies—ranging from targeted microglial modulation and cell‐free exosomes to gene therapy—that aim to restore niche function [318]. As these powerful interventions emerge, they bring a complex landscape of ethical and societal challenges, demanding proactive deliberation on issues of patient safety, equity, and public policy to ensure responsible translation (Figure 3) [319].

### 6.1. Advanced Imaging and Labeling Strategies

Breakthroughs in imaging have been pivotal in revealing the intricate crosstalk within the niche. Intravital multiphoton microscopy enables longitudinal tracking of interactions between immune cells, glia, neurons, and the vasculature in living systems [315]. Three‐photon microscopy has enabled noninvasive deep‐brain imaging of the mouse SVZ, detecting direct NSC‐vasculature interactions [320]. Complementing multiphoton microscopy are other modalities like super‐resolution microscopy and PET, while the integration of AI is enhancing the interpretation of imaging data [321, 322]. The power of these techniques is magnified by sophisticated genetic labeling strategies, including multicolor reporters like Brainbow and CRISPR‐based tools that allow for efficient gene targeting and real‐time cellular tracking in NSC research [316, 323].

### 6.2. Omics and Systems Biology Approaches

The convergence of high‐throughput omics with systems biology provides a holistic understanding of the niche. scRNA‐seq has revolutionized the characterization of cellular heterogeneity, revealing that NSCs exist as a complex continuum rather than discrete populations [324]. Spatial omics methodologies add a critical layer of contextual information, allowing for the analysis of cell–cell interactions in their native tissue architecture [325]. Proteomic analyses offer a lens to examine functional changes during aging, revealing widespread alterations in immune proteins and the stoichiometry of essential protein complexes, leading to the concept of a “proteomic aging clock”—a computational model that estimates biological age based on age‐associated patterns in protein expression, offering insights into systemic aging and potential biomarkers for age‐related diseases [326, 327]. Computational models are indispensable for integrating these vast datasets to simulate niche dynamics and predict therapeutic outcomes [328].

### 6.3. Novel Therapeutic Strategies

Recent advances have spurred the development of interventions aimed at modulating the core components of the niche. However, these strategies exist on a spectrum of maturity, ranging from robust preclinical mechanisms to emerging experimental paradigms that require rigorous validation.

#### 6.3.1. Pharmacological Modulation of Glial States (Robust Preclinical Evidence)

The most advanced strategies target the inflammatory state of the niche. A prominent approach involves the targeted depletion of microglia using CSF1R inhibitors (e.g., PLX3397 and PLX5622), followed by repopulation with homeostatic microglia. While this “reset” strategy has shown promise in reducing neuroinflammation and improving behavioral outcomes in animal models of AD, PD, and multiple sclerosis [329], the translation is complex. Critical limitations exist: complete microglial depletion in mice exacerbates injury severity and impairs functional recovery in spinal cord injury models, disrupting glial scar formation, enhancing immune cell infiltration, and reducing neuronal survival [330]. Furthermore, the long‐term effects of depletion on astrocyte and oligodendrocyte crosstalk remain functionally ambiguous [329]. Parallel to depletion is the selective elimination of senescent cells. The accumulation of p16^Ink4a+^ senescent microglia in the aging hippocampus drives cognitive decline via a SASP [331]. Proof‐of‐concept studies demonstrate that senolytic interventions (e.g., Dasatinib plus Quercetin or ABT‐737) can selectively eliminate these senescent microglia, thereby reducing the hyper‐phagocytosis of excitatory synapses and restoring long‐term potentiation and cognitive function in aged and LPS‐induced inflammatory models [332].

#### 6.3.2. Bioengineering and Delivery Systems

To overcome the BBB, novel delivery systems are being engineered to physically bridge niche components. Immunomodulatory hydrogel microspheres (e.g., MP/RIL4) have been developed to mechanically and chemically link microglia with the neurovascular unit. In ischemic stroke models, these microspheres successfully upregulated anti‐inflammatory factors (IL‐10 and Arg‐1) while downregulating pro‐inflammatory markers (IL‐1β), effectively orchestrating the immune‐neurovascular crosstalk to promote angiogenesis and neurogenesis [333]. Intranasal delivery represents a noninvasive route to bypass the BBB, allowing for the direct transport of growth factors, stem cells, and exosomes to the CNS to treat neuroinflammation in AD and stroke [334]. Concurrently, cell‐free therapeutics such as MSC‐derived exosomes are emerging as a “new remedy” to attenuate neuroinflammation and induce neurogenesis without the risks of cell transplantation, though standardization of cargo remains a hurdle [318].

#### 6.3.3. Metabolic and Genetic Interventions

Beyond direct niche manipulation, systemic and genetic strategies offer broader modulation. Calorie restriction and intermittent fasting have been shown to dampen systemic inflammatory mediators (e.g., TNF‐α and IL‐6) and may promote osteoprogenitor cells, potentially preserving the niche through metabolic regulation, although human adherence remains a challenge [335]. Drug repurposing also shows potential; the combination of lovastatin and selegiline has demonstrated a synergistic effect on the differentiation of bone marrow stromal cells into neuron‐like cells via increased expression of nestin and NF‐68, optimizing stem cell therapeutic approaches [336]. Finally, gene therapy utilizing AAV vectors to deliver neurotrophic factors (e.g., BDNF and GDNF) has moved into clinical trials for AD and PD, though ensuring long‐term safety and efficacy regarding serotypes and administration routes continues to be a primary focus of ongoing research [337].

### 6.4. Ethical and Societal Implications

Advancements in manipulating the neurogenic niche usher in a complex landscape of ethical, legal, and societal challenges. Stem cell‐based strategies raise concerns about patient safety, the potential for exploitation through unproven treatments, and long‐term risks such as tumorigenesis, demanding robust regulatory oversight [338]. A critical societal challenge is ensuring equitable access to these interventions. Social, economic, and environmental factors are powerful determinants of brain health, and translational research must integrate equity as a primary goal to avoid creating new frontiers in health disparities [339]. The prospect of restoring neurogenesis to extend cognitive healthspan necessitates a paradigm shift in public health policy toward proactive, preventive care while ensuring that the social determinants of health are addressed to safeguard public welfare as these powerful new technologies emerge [339].

#### 6.4.1. Unresolved Questions: The Double‐Edged Sword of Niche Manipulation

While strategies to reset the neurogenic niche via cellular elimination offer profound disease‐modifying potential, they introduce a critical double‐edged sword regarding tissue structural integrity and acute injury response. The pharmacological depletion of microglia (e.g., via CSF1R inhibitors like PLX5622) has demonstrated efficacy in specific chronic models by eliminating maladaptive, pro‐inflammatory populations [340, 341]. However, this intervention reveals a dangerous vulnerability: microglia are essential for ’containment’ functions. In Alzheimer’s models, depletion disrupts the compaction of amyloid plaques, leading to enhanced neuritic dystrophy [342]. Furthermore, during acute CNS injury, such as spinal cord injury or stroke, microglia orchestrate the formation of the protective glial scar [343, 344]. Depletion during these critical windows removes the ’brakes’ on astrocyte activation, leading to disrupted scar organization, a paradoxical surge in inflammatory cytokines, and widespread lesion expansion [330, 345, 346].

Parallel to this is the unresolved structural risk of senolytic therapies. While clearing senescent cells is intended to ameliorate the toxic SASP and has shown promise in restoring BBB integrity in aged mice [347, 348], a theoretical concern persists regarding the creation of structural gaps. Because pericytes and endothelial cells physically comprise the BBB, their rapid elimination raises the risk of transient barrier collapse before regeneration can occur [349, 350]. Indeed, the acute loss of pericytes—even if dysfunctional—has been shown to trigger rapid circulatory failure, loss of neurotrophic support (e.g., Pleiotrophin), and subsequent neuronal death [351]. Thus, the long‐term safety of culling nonregenerative structural populations remains a significant frontier that must be resolved, balancing the removal of the SASP against the maintenance of physical barrier continuity.

## 7. Discussion

The adult neurogenic niche demonstrates the brain’s enduring capacity for plasticity, facilitated by a dynamic collaboration of vascular, glial, and NSCs. While this review has outlined the cooperative architecture of this system, synthesizing the current literature reveals significant knowledge gaps, particularly regarding the translatability of rodent mechanisms to human physiology and the paradoxical risks associated with therapeutic manipulation of the niche.

The most profound conflict in the field remains the persistence of AHN in humans. The literature is currently divided between findings suggesting AHN serves as a robust mechanism for plasticity throughout life [18, 258] and contradictory reports indicating a sharp cessation of neurogenesis in childhood [235]. This dichotomy does not appear to stem from biological variability, but rather from a methodological crisis. Critical analysis suggests that standard histological practices—specifically prolonged fixation and long PMIs—create a “false negative” landscape by masking labile epitopes like DCX [236, 240]. Furthermore, the accumulation of lipofuscin in the aging human brain generates autofluorescence that confounds signal detection, leading to potential misinterpretations of glial cells as neurons [244, 261]. Recent breakthroughs utilizing single‐nucleus RNA sequencing have begun to resolve this debate by identifying transcriptomic signatures of proliferating neuroblasts that bypass histological artifacts [247]. However, a key knowledge gap remains: human immature neurons exhibit a protracted maturation profile distinct from the rapid cycles observed in rodents [229, 248]. This suggests that the human niche may function less as a factory for high‐throughput replacement and more as a reservoir of suspended plasticity. Future research is important to prioritize spatial transcriptomics to map these cells within their native architecture to definitively validate their functional integration [252, 264]. Beyond the existence of neurogenesis, the functional implication of manipulating the niche reveals a complex causal landscape where interventions can induce paradoxical toxicity. While targeting the niche offers disease‐modifying potential, current evidence highlights a double‐edged sword. Pharmacological elimination of microglia (e.g., via CSF1R inhibitors) has shown promise in clearing amyloid plaques in Alzheimer’s models [340]. However, conflicting evidence from acute injury models demonstrates that this strategy risks exacerbating damage. In spinal cord injury and stroke, microglial depletion disrupts the formation of the protective glial scar, leading to lesion expansion and impaired recovery [330, 345]. This suggests that the “neurotoxic” phenotype is context‐dependent, and broad ablation removes essential containment functions. Similarly, the use of senolytics to clear senescent cells presents an unresolved structural risk. While clearing senescent cells alleviates the SASP [331], the removal of senescent endothelial cells or pericytes—which physically comprise the BBB—could theoretically precipitate transient barrier collapse or micro‐hemorrhages before regeneration occurs [349, 351].

Furthermore, a critical synthesis of the literature reveals that an active niche is not inherently beneficial. In pathological contexts, the mechanisms of neurogenesis can be subverted to drive disease, a concept termed maladaptive plasticity. In TLE, seizure activity stimulates the niche to produce ectopic granule cells that migrate aberrantly and integrate into excitatory loops, actively promoting hyperexcitability rather than repair [302, 303]. Similarly, in AD, new neurons may act as a vulnerable substrate for Tau pathology, described as the Trojan horse hypothesis, or remain in a stalled, immature state that contributes to network noise rather than memory encoding [309, 311]. This challenges the simplistic therapeutic goal of boosting neurogenesis; effective translation requires not just increasing proliferation but restoring the guidance cues (e.g., Reelin and GABAergic tone) that ensure correct integration [66, 303]. Finally, the integration of systemic signals via the gut–brain axis offers a noninvasive therapeutic avenue, yet the causal mechanisms in humans remain under‐defined. While psychobiotics robustly modulate neurogenesis in rodents via the vagus nerve and SCFAs [206, 215], human trials suffer from inconsistency due to a lack of strain specificity [218]. Moving forward, the field needs to transition from descriptive phenomenology to mechanistic validation. This requires the application of multiomics and 4D intravital imaging to visualize the niche in real‐time [320, 325]. Ultimately, harnessing the neurogenic niche requires a precision medicine approach that accounts for the delicate balance between promoting plasticity and maintaining structural homeostasis, ensuring that interventions do not inadvertently dismantle the very architecture they aim to repair.

## Funding

This research received no specific grant from any funding agency in the public, commercial, or not‐for‐profit sectors.

## Conflicts of Interest

The author declares no conflicts of interest.

## Data Availability

The data that support the findings of this study are openly available in Authorea at https://doi.org/10.22541/au.176081250.01618371/v1.

## References

[1] Kempermann G. , What Is Adult Hippocampal Neurogenesis Good for?, Frontiers in Neuroscience. (2022) 16, 852680. 35495058 10.3389/fnins.2022.852680 PMC9051245

[2] McHugh S. B. , Lopes-dos-Santos V. , and Gava G. P. , et al.Adult-Born Dentate Granule Cells Promote Hippocampal Population Sparsity, Nature Neuroscience. (2022) 25, no. 11, 1481–1491, 10.1038/s41593-022-01176-5. 36216999 PMC9630129

[3] Wang Z. , Yang K. , and Sun X. , Effect of Adult Hippocampal Neurogenesis on Pattern Separation and Its Applications, Cognitive Neurodynamics. (2024) 18, no. 5, 1–14, 10.1007/s11571-024-10110-3. PMC11564429 39568526

[4] Wang M. , van Bruggen R. , Mohammed L. , Egor K. , and Tan Q. , Loss of NFIA Impairs Adult Hippocampal Neurogenesis, Hippocampus. (2025) 35, no. 4, 10.1002/hipo.70016, e70016. 40459218 PMC12131691

[5] Petkova S. P. , Pride M. , and Klocke C. , et al.Cyclin D2-Knock-Out Mice with Attenuated Dentate Gyrus Neurogenesis Have Robust Deficits in Long-Term Memory Formation, Scientific Reports. (2020) 10, no. 1, 10.1038/s41598-020-65090-1, 8204. 32424171 PMC7235216

[6] Jones K. L. , Zhou M. , and Jhaveri D. J. , Dissecting the Role of Adult Hippocampal Neurogenesis Towards Resilience Versus Susceptibility to Stress-Related Mood Disorders, npj Science of Learning. (2022) 7, no. 1, 10.1038/s41539-022-00133-y, 16. 35842419 PMC9288448

[7] Hovorka M. , Ewing D. , and Middlemas D. S. , Chronic SSRI Treatment, but Not Norepinephrine Reuptake Inhibitor Treatment, Increases Neurogenesis in Juvenile Rats, International Journal of Molecular Sciences. (2022) 23, no. 13, 10.3390/ijms23136919, 6919. 35805924 PMC9267057

[8] Kondo M. , Molecular Mechanisms of Exercise-induced Hippocampal Neurogenesis and Antidepressant Effects, JMA Journal. (2023) 6, no. 2, 114–119, 10.31662/jmaj.2023-0010. 37179732 PMC10169258

[9] Planchez B. , Lagunas N. , and Le Guisquet A.-M. , et al.Increasing Adult Hippocampal Neurogenesis Promotes Resilience in a Mouse Model of Depression, Cells. (2021) 10, no. 5, 10.3390/cells10050972, 972. 33919292 PMC8143348

[10] Leschik J. , Lutz B. , and Gentile A. , Stress-Related Dysfunction of Adult Hippocampal Neurogenesis—An Attempt for Understanding Resilience?, International Journal of Molecular Sciences. (2021) 22, no. 14, 10.3390/ijms22147339, 7339. 34298958 PMC8305135

[11] Bonafina A. , Paratcha G. , and Ledda F. , Deciphering New Players in the Neurogenic Adult Hippocampal Niche, Frontiers in Cell and Developmental Biology. (2020) 8, 10.3389/fcell.2020.00548, 548. 32714932 PMC7346873

[12] Li Y. and Guo W. , Neural Stem Cell Niche and Adult Neurogenesis, The Neuroscientist. (2021) 27, no. 3, 235–245, 10.1177/1073858420939034. 32729779

[13] Pérez-Domínguez M. , Tovar-y-Romo L. B. , and Zepeda A. , Neuroinflammation and Physical Exercise as Modulators of Adult Hippocampal Neural Precursor Cell Behavior, Reviews in the Neurosciences. (2017) 29, no. 1, 1–20, 10.1515/revneuro-2017-0024, 2-s2.0-85037659129. 28873068

[14] Obernier K. and Alvarez-Buylla A. , Neural Stem Cells: Origin, Heterogeneity and Regulation in the Adult Mammalian Brain, Development. (2019) 146, no. 4, 10.1242/dev.156059, 2-s2.0-85061845529, dev156059. 30777863 PMC6398449

[15] Diaz-Aparicio I. , Paris I. , and Sierra-Torre V. , et al.Microglia Actively Remodel Adult Hippocampal Neurogenesis Through the Phagocytosis Secretome, The Journal of Neuroscience. (2020) 40, no. 7, 1453–1482, 10.1523/JNEUROSCI.0993-19.2019. 31896673 PMC7044727

[16] Kjell J. , Fischer-Sternjak J. , and Thompson A. J. , et al.Defining the Adult Neural Stem Cell Niche Proteome Identifies Key Regulators of Adult Neurogenesis, Cell Stem Cell. (2020) 26, no. 2, 277–293.e8, e8 10.1016/j.stem.2020.01.002. 32032526 PMC7005820

[17] Kerever A. and Arikawa-Hirasawa E. , Optimal Extracellular Matrix Niches for Neurogenesis: Identifying Glycosaminoglycan Chain Composition in the Subventricular Neurogenic Zone, Frontiers in Neuroanatomy. (2021) 15, 10.3389/fnana.2021.764458, 764458. 34671246 PMC8520954

[18] Moreno-Jiménez E. P. , Terreros-Roncal J. , Flor-García M. , Rábano A. , and Llorens-Martín M. , Evidences for Adult Hippocampal Neurogenesis in Humans, The Journal of Neuroscience. (2021) 41, no. 12, 2541–2553, 10.1523/JNEUROSCI.0675-20.2020. 33762406 PMC8018741

[19] Simard S. , Matosin N. , and Mechawar N. , Adult Hippocampal Neurogenesis in the Human Brain: Updates, Challenges, and Perspectives, The Neuroscientist. (2025) 31, no. 2, 141–158, 10.1177/10738584241252581. 38757781

[20] Terstege D. J. , Addo-Osafo K. , Campbell Teskey G. , and Epp J. R. , New Neurons in Old Brains: Implications of Age in the Analysis of Neurogenesis in Post-Mortem Tissue, Molecular Brain. (2022) 15, no. 1, 10.1186/s13041-022-00926-7, 38. 35501905 PMC9063342

[21] Charvet C. J. and Finlay B. L. , Comparing Adult Hippocampal Neurogenesis Across Species: Translating Time to Predict the Tempo in Humans, Frontiers in Neuroscience. (2018) 12, 10.3389/fnins.2018.00706, 2-s2.0-85055285087, 706. 30344473 PMC6182078

[22] Jahanbin K. , The Neurogenic Niche: Interactions Among Vessels, Glia, and Neural Stem Cells, 2025, Authorea Preprints, 10.22541/au.176081250.01618371/v1. PMC13071373 41983237

[23] Ottone C. and Parrinello S. , Multifaceted Control of Adult SVZ Neurogenesis by the Vascular Niche, Cell Cycle. (2015) 14, no. 14, 2222–2225, 10.1080/15384101.2015.1049785, 2-s2.0-84943744728. 26115376 PMC4612675

[24] Segarra M. , Aburto M. R. , Hefendehl J. , and Acker-Palmer A. , Neurovascular Interactions in the Nervous System, Annual Review of Cell and Developmental Biology. (2019) 35, no. 1, 615–635, 10.1146/annurev-cellbio-100818-125142, 2-s2.0-85072965130. 31590587

[25] Wang W. , Su L. , Wang Y. , Li C. , Ji F. , and Jiao J. , Endothelial Cells Mediated by UCP2 Control the Neurogenic-to-Astrogenic Neural Stem Cells Fate Switch During Brain Development, Advanced Science. (2022) 9, no. 18, 10.1002/advs.202105208, 2105208. 35488517 PMC9218656

[26] Wang Y. , Su L. , and Wang W. , et al.Endothelial Arid1A Deletion Disrupts the Balance Among Angiogenesis, Neurogenesis and Gliogenesis in the Developing Brain, Cell Proliferation. (2023) 56, no. 5, 10.1111/cpr.13447, e13447. 36916004 PMC10212716

[27] Benarroch E. , What Are the Roles of Pericytes in the Neurovascular Unit and Its Disorders?, Neurology. (2023) 100, no. 20, 970–977, 10.1212/WNL.0000000000207379. 37188542 PMC10186232

[28] Procter T. V. , Williams A. , and Montagne A. , Interplay Between Brain Pericytes and Endothelial Cells in Dementia, The American Journal of Pathology. (2021) 191, no. 11, 1917–1931, 10.1016/j.ajpath.2021.07.003. 34329605 PMC12179519

[29] Calvo C.-F. , Fontaine R. H. , and Soueid J. , et al.Vascular Endothelial Growth Factor Receptor 3 Directly Regulates Murine Neurogenesis, Genes & Development. (2011) 25, no. 8, 831–844, 10.1101/gad.615311, 2-s2.0-79955408372. 21498572 PMC3078708

[30] During M. J. and Cao L. , VEGF, A Mediator of the Effect of Experience on Hippocampal Neurogenesis, Current Alzheimer Research. (2006) 3, no. 1, 29–33, 10.2174/156720506775697133, 2-s2.0-33645865549. 16472200

[31] Marie C. , Pedard M. , and Quirie A. , et al.Brain-Derived Neurotrophic Factor Secreted by the Cerebral Endothelium: A New Actor of Brain Function?, Journal of Cerebral Blood Flow & Metabolism. (2018) 38, no. 6, 935–949, 10.1177/0271678X18766772, 2-s2.0-85045050533. 29557702 PMC5998997

[32] Shen Q. , Wang Y. , and Kokovay E. , et al.Adult SVZ Stem Cells Lie in a Vascular Niche: A Quantitative Analysis of Niche Cell-Cell Interactions, Cell Stem Cell. (2008) 3, no. 3, 289–300, 10.1016/j.stem.2008.07.026, 2-s2.0-50849142558. 18786416 PMC2747473

[33] Sato Y. , Kiyozumi D. , and Futaki S. , et al.Ventricular–Subventricular Zone Fractones Are Speckled Basement Membranes That Function as a Neural Stem Cell Niche, Molecular Biology of the Cell. (2019) 30, no. 1, 56–68, 10.1091/mbc.E18-05-0286, 2-s2.0-85059253087. 30379609 PMC6337917

[34] Apple D. M. and Kokovay E. , Vascular Niche Contribution to Age-Associated Neural Stem Cell Dysfunction, American Journal of Physiology-Heart and Circulatory Physiology. (2017) 313, no. 5, H896–H902, 10.1152/ajpheart.00154.2017, 2-s2.0-85032922516. 28801522 PMC5792207

[35] Santisteban M. M. and Iadecola C. , The Pathobiology of Neurovascular Aging, Neuron. (2025) 113, no. 1, 49–70, 10.1016/j.neuron.2024.12.014. 39788087 PMC12136575

[36] Soto-Rojas L. O. , Pacheco-Herrero M. , and Martínez-Gómez P. A. , et al.The Neurovascular Unit Dysfunction in Alzheimer’s Disease, International Journal of Molecular Sciences. (2021) 22, no. 4, 10.3390/ijms22042022, 2022. 33670754 PMC7922832

[37] Zipfel G. J. , Han H. , Ford A. L. , and Lee J.-M. , Cerebral Amyloid Angiopathy, Stroke. (2009) 40, no. 3_suppl_1, S16–S9, 10.1161/STROKEAHA.108.533174, 2-s2.0-62949218521. 19064794 PMC2680011

[38] Morris A. W. , Carare R. O. , Schreiber S. , and Hawkes C. A. , The Cerebrovascular Basement Membrane: Role in the Clearance of β-Amyloid and Cerebral Amyloid Angiopathy, Frontiers in Aging Neuroscience. (2014) 6, 10.3389/fnagi.2014.00251, 2-s2.0-84907222721, 251. 25285078 PMC4168721

[39] Badanjak K. , Fixemer S. , Smajić S. , Skupin A. , and Grünewald A. , The Contribution of Microglia to Neuroinflammation in Parkinson’s Disease, International Journal of Molecular Sciences. (2021) 22, no. 9, 10.3390/ijms22094676, 4676. 33925154 PMC8125756

[40] Paul G. and Elabi O. F. , Microvascular Changes in Parkinson’s Disease- Focus on the Neurovascular Unit, Frontiers in Aging Neuroscience. (2022) 14, 10.3389/fnagi.2022.853372, 853372. 35360216 PMC8960855

[41] Schneider J. , Karpf J. , and Beckervordersandforth R. , Role of Astrocytes in the Neurogenic Niches, Methods and Protocols. (2019) 1938, 19–33, 10.1007/978-1-4939-9068-9. 30617970

[42] Gage F. H. , Molecular and Cellular Mechanisms Contributing to the Regulation, Proliferation and Differentiation of Neural Stem Cells in the Adult Dentate Gyrus, The Keio Journal of Medicine. (2010) 59, no. 3, 79–83, 10.2302/kjm.59.79, 2-s2.0-78249267229. 20881448

[43] Kim Y. , Dube S. E. , and Park C. B. , Brain Energy Homeostasis: The Evolution of the Astrocyte-Neuron Lactate Shuttle Hypothesis, The Korean Journal of Physiology & Pharmacology. (2025) 29, no. 1, 1–8, 10.4196/kjpp.24.388. 39725609 PMC11694005

[44] Wu A. , Lee D. , and Xiong W.-C. , Lactate Metabolism, Signaling, and Function in Brain Development, Synaptic Plasticity, Angiogenesis, and Neurodegenerative Diseases, International Journal of Molecular Sciences. (2023) 24, no. 17, 10.3390/ijms241713398, 13398. 37686202 PMC10487923

[45] Coulter D. A. and Eid T. , Astrocytic Regulation of Glutamate Homeostasis in Epilepsy, Glia. (2012) 60, no. 8, 1215–1226, 10.1002/glia.22341, 2-s2.0-84862321615. 22592998 PMC3375386

[46] Peterson A. R. and Binder D. K. , Astrocyte Glutamate Uptake and Signaling as Novel Targets for Antiepileptogenic Therapy, Frontiers in Neurology. (2020) 11, 10.3389/fneur.2020.01006, 1006. 33013665 PMC7505989

[47] Wang H. , Yan M. , and Cheng Z. , et al.WNT Signaling Influences Neurological Function and Psychiatric Disorders Through Regulating Glia Phenotypes and Neuron Plasticity, 2021.

[48] Ashton R. S. , Conway A. , and Pangarkar C. , et al.Astrocytes Regulate Adult Hippocampal Neurogenesis Through Ephrin-B Signaling, Nature Neuroscience. (2012) 15, no. 10, 1399–1406, 10.1038/nn.3212, 2-s2.0-84866731826. 22983209 PMC3458152

[49] Christopherson K. S. , Ullian E. M. , and Stokes C. C. , et al.Thrombospondins Are Astrocyte-Secreted Proteins That Promote CNS Synaptogenesis, Cell. (2005) 120, no. 3, 421–433, 10.1016/j.cell.2004.12.020, 2-s2.0-13544273916. 15707899

[50] Wang F.-W. , Hao H.-B. , and Zhao S.-D. , et al.Roles of Activated Astrocyte in Neural Stem Cell Proliferation and Differentiation, Stem Cell Research. (2011) 7, no. 1, 41–53, 10.1016/j.scr.2011.03.004, 2-s2.0-79956341465. 21530437

[51] Sofroniew M. V. , Molecular Dissection of Reactive Astrogliosis and Glial Scar Formation, Trends in Neurosciences. (2009) 32, no. 12, 638–647, 10.1016/j.tins.2009.08.002, 2-s2.0-70449678738. 19782411 PMC2787735

[52] Chinta S. J. , Woods G. , Rane A. , Demaria M. , Campisi J. , and Andersen J. K. , Cellular Senescence and the Aging Brain, Experimental Gerontology. (2015) 68, 3–7, 10.1016/j.exger.2014.09.018, 2-s2.0-84941316442. 25281806 PMC4382436

[53] Jha M. K. , Jo M. , Kim J.-H. , and Suk K. , Microglia-Astrocyte Crosstalk: An Intimate Molecular Conversation, The Neuroscientist. (2019) 25, no. 3, 227–240, 10.1177/1073858418783959, 2-s2.0-85048927473. 29931997

[54] Cunningham C. L. , Martínez-Cerdeño V. , and Noctor S. C. , Microglia Regulate the Number of Neural Precursor Cells in the Developing Cerebral Cortex, The Journal of Neuroscience. (2013) 33, no. 10, 4216–4233, 10.1523/JNEUROSCI.3441-12.2013, 2-s2.0-84874586343. 23467340 PMC3711552

[55] Ueno M. , Fujita Y. , and Tanaka T. , et al.Layer V Cortical Neurons Require Microglial Support for Survival During Postnatal Development, Nature Neuroscience. (2013) 16, no. 5, 543–551, 10.1038/nn.3358, 2-s2.0-84876939061. 23525041

[56] Rusin D. , Vahl Becirovic L. , and Lyszczarz G. , et al.Microglia-Derived Insulin-Like Growth Factor 1 Is Critical for Neurodevelopment, Cells. (2024) 13, no. 2, 10.3390/cells13020184, 184. 38247874 PMC10813844

[57] Yu D. , Jain S. , and Wangzhou A. , et al.Microglia Regulate GABAergic Neurogenesis in Prenatal Human Brain Through IGF1, Nature. (2025) 646, no. 8085, 676–686, 10.1038/s41586-025-09362-8. 40770097 PMC12527950

[58] Chen Z. and Palmer T. D. , Differential Roles of TNFR1 and TNFR2 Signaling in Adult Hippocampal Neurogenesis, Brain, Behavior, and Immunity. (2013) 30, 45–53, 10.1016/j.bbi.2013.01.083, 2-s2.0-84876883238. 23402793 PMC3641155

[59] Green H. F. , Treacy E. , Keohane A. K. , Sullivan A. M. , O’Keeffe G. W. , and Nolan Y. M. , A Role for Interleukin-1β in Determining the Lineage Fate of Embryonic Rat Hippocampal Neural Precursor Cells, Molecular and Cellular Neuroscience. (2012) 49, no. 3, 311–321, 10.1016/j.mcn.2012.01.001, 2-s2.0-84856795338. 22270046

[60] Cornejo F. and von Bernhardi R. , Age-Dependent Changes in the Activation and Regulation of Microglia, Glial Cells in Health and Disease of the CNS, 2016, Springer International Publishing, 205–226. 10.1007/978-3-319-40764-7_10 27714691

[61] Xu Y.-J. , Au N. P. B. , and Ma C. H. E. , Functional and Phenotypic Diversity of Microglia: Implication for Microglia-Based Therapies for Alzheimer’s Disease, Frontiers in Aging Neuroscience. (2022) 14, 10.3389/fnagi.2022.896852, 896852. 35693341 PMC9178186

[62] Sarapultsev A. , Gusev E. , Chereshnev V. , Komelkova M. , and Hu D. , Neuroimmune Interactions in Stress and Depression: Exploring the Molecular and Cellular Mechanisms Within the Neuroinflammation-Depression Nexus, Current Medicinal Chemistry. (2024) 31, 10.2174/0109298673320710240920055041. 39350557

[63] Jain N. , Lewis C. A. , Ulrich J. D. , and Holtzman D. M. , Chronic TREM2 Activation Exacerbates Aβ-Associated Tau Seeding and Spreading, Journal of Experimental Medicine. (2023) 220, no. 1, 10.1084/jem.20220654, e20220654. 36219197 PMC9559604

[64] Valiukas Z. , Tangalakis K. , Apostolopoulos V. , and Feehan J. , Microglial Activation States and Their Implications for Alzheimer’s Disease, The Journal of Prevention of Alzheimer’s Disease. (2025) 12, no. 1, 10.1016/j.tjpad.2024.100013, 100013. PMC12184064 39800461

[65] Song J. , Zhong C. , and Bonaguidi M. A. , et al.Neuronal Circuitry Mechanism Regulating Adult Quiescent Neural Stem-Cell Fate Decision, Nature. (2012) 489, no. 7414, 150–154, 10.1038/nature11306, 2-s2.0-84865801305. 22842902 PMC3438284

[66] Bao H. , Asrican B. , and Li W. , et al.Long-Range GABAergic Inputs Regulate Neural Stem Cell Quiescence and Control Adult Hippocampal Neurogenesis, Cell Stem Cell. (2017) 21, no. 5, 604–617.e5, 10.1016/j.stem.2017.10.003, 2-s2.0-85043527961. 29100013 PMC5689456

[67] Shors T. J. , Anderson M. L. , Curlik Ii D. , and Nokia M. S. , Use It or Lose It: How Neurogenesis Keeps the Brain Fit for Learning, Behavioural Brain Research. (2012) 227, no. 2, 450–458, 10.1016/j.bbr.2011.04.023, 2-s2.0-84855967801. 21536076 PMC3191246

[68] Tashiro A. , Sandler V. M. , Toni N. , Zhao C. , and Gage F. H. , NMDA-Receptor-Mediated, Cell-Specific Integration of New Neurons in Adult Dentate Gyrus, Nature. (2006) 442, no. 7105, 929–933, 10.1038/nature05028, 2-s2.0-33747827189. 16906136

[69] Vivar C. , Potter M. C. , and Choi J. , et al.Monosynaptic Inputs to New Neurons in the Dentate Gyrus, Nature Communications. (2012) 3, no. 1, 10.1038/ncomms2101, 2-s2.0-84869384487, 1107. PMC4603575 23033083

[70] Campbell N. R. , Fernandes C. C. , Halff A. W. , and Berg D. K. , Endogenous Signaling Through 0A0;7-Containing Nicotinic Receptors Promotes Maturation and Integration of Adult-Born Neurons in the Hippocampus, Journal of Neuroscience. (2010) 30, no. 26, 8734–8744, 10.1523/JNEUROSCI.0931-10.2010, 2-s2.0-77954408745. 20592195 PMC2905643

[71] Jessberger S. and Parent J. M. , Epilepsy and Adult Neurogenesis, Cold Spring Harbor Perspectives in Biology. (2015) 7, no. 12, 10.1101/cshperspect.a020677, 2-s2.0-84949266866, a020677. 26552418 PMC4665072

[72] Willie C. K. , Cowan E. C. , and Ainslie P. N. , et al.Neurovascular Coupling and Distribution of Cerebral Blood Flow During Exercise, Journal of Neuroscience Methods. (2011) 198, no. 2, 270–273, 10.1016/j.jneumeth.2011.03.017, 2-s2.0-79957712597. 21459113

[73] Ci C. , Moon H. Y. , and Van Praag H. , On the Run for Hippocampal Plasticity, Cold Spring Harbor Perspectives in Medicine. (2018) 8, no. 4, 10.1101/cshperspect.a029736, 2-s2.0-85031303354, a029736. 28495803 PMC5880155

[74] O’Riordan K. J. , Collins M. K. , and Moloney G. M. , et al.Short Chain Fatty Acids: Microbial Metabolites for Gut-Brain Axis Signalling, Molecular and Cellular Endocrinology. (2022) 546, 10.1016/j.mce.2022.111572, 111572. 35066114

[75] Carabotti M. , Scirocco A. , Maselli M. A. , and Severi C. , The Gut-Brain Axis: Interactions Between Enteric Microbiota, Central and Enteric Nervous Systems, Annals of Gastroenterology: Quarterly Publication of the Hellenic Society of Gastroenterology. (2015) 28, no. 2, 203. PMC4367209 25830558

[76] Wrann C. D. , White J. P. , and Salogiannnis J. , et al.Exercise Induces Hippocampal BDNF Through a PGC-1α/FNDC5 Pathway, Cell Metabolism. (2013) 18, no. 5, 649–659, 10.1016/j.cmet.2013.09.008, 2-s2.0-84887468128. 24120943 PMC3980968

[77] Xu H. , Tian X. , and Wang Y. , et al.Exercise Promotes Hippocampal Neurogenesis in T2DM Mice via Irisin/TLR4/MyD88/NF-κB-Mediated Neuroinflammation Pathway, Biology. (2024) 13, no. 10, 10.3390/biology13100809, 809. 39452118 PMC11504848

[78] Huang B. , Chen K. , and Li Y. , Aerobic Exercise, an Effective Prevention and Treatment for Mild Cognitive Impairment, Frontiers in Aging Neuroscience. (2023) 15, 10.3389/fnagi.2023.1194559, 1194559. 37614470 PMC10442561

[79] Goekce E. and Gün N. , The Relationship Between Exercise, Cathepsin B, and Cognitive Functions: Systematic Review, Perceptual and Motor Skills. (2023) 130, no. 4, 1366–1385, 10.1177/00315125231176980. 37202717

[80] Jayaraj K. , Kumar R. , Shyamasundar S. , Arumugam T. V. , Polepalli J. S. , and Dheen S. T. , Spatial Transcriptomic Analysis Reveals HDAC Inhibition Modulates Microglial Dynamics to Protect Against Ischemic Stroke in Mice, Glia. (2025) 73, no. 9, 1817–1840, 10.1002/glia.70035. 40415727 PMC12313008

[81] Wei H. , Ren Y. , and Yu C. , et al.Sodium Butyrate Alleviates Chronic Alcoholic Neuroinflammation by Regulating Microgila Polarization Through GPR109A/PPAR-γ/NF-κB Signaling Pathway, 2022.

[82] Thomson C. A. , McColl A. , Graham G. J. , and Cavanagh J. , Sustained Exposure to Systemic Endotoxin Triggers Chemokine Induction in the Brain Followed by a Rapid Influx of Leukocytes, Journal of Neuroinflammation. (2020) 17, no. 1, 10.1186/s12974-020-01759-8, 94. 32213184 PMC7098135

[83] Sumi N. , Nishioku T. , and Takata F. , et al.Lipopolysaccharide-Activated Microglia Induce Dysfunction of the Blood–Brain Barrier in Rat Microvascular Endothelial Cells Co-Cultured With Microglia, Cellular and Molecular Neurobiology. (2010) 30, no. 2, 247–253, 10.1007/s10571-009-9446-7, 2-s2.0-77952095680. 19728078 PMC11498813

[84] Nishioku T. , Dohgu S. , and Takata F. , et al.Detachment of Brain Pericytes From the Basal Lamina is Involved in Disruption of the Blood–Brain Barrier Caused by Lipopolysaccharide-Induced Sepsis in Mice, Cellular and Molecular Neurobiology. (2009) 29, no. 3, 309–316, 10.1007/s10571-008-9322-x, 2-s2.0-67349280877. 18987969 PMC11506181

[85] Paul S. N. , Wingenfeld K. , Otte C. , and Meijer O. C. , Brain Mineralocorticoid Receptor in Health and Disease: From Molecular Signalling to Cognitive and Emotional Function, British Journal of Pharmacology. (2022) 179, no. 13, 3205–3219, 10.1111/bph.15835. 35297038 PMC9323486

[86] Herting M. , Cotter D. , and Ahmadi H. , et al.Sex-Specific Effects in How Childhood Exposures to Multiple Ambient Air Pollutants Affect White Matter Microstructure Development Across Early Adolescence, Research Square. (2023) 3.

[87] Boda E. , Rigamonti A. E. , and Bollati V. , Understanding the Effects of Air Pollution on Neurogenesis and Gliogenesis in the Growing and Adult Brain, Current Opinion in Pharmacology. (2020) 50, 61–66, 10.1016/j.coph.2019.12.003. 31896533

[88] Guo S. , Wang H. , and Yin Y. , Microglia Polarization From M1 to M2 in Neurodegenerative Diseases, Frontiers in Aging Neuroscience. (2022) 14, 10.3389/fnagi.2022.815347, 815347. 35250543 PMC8888930

[89] Makino H. , Top-Down Control: A Unified Principle of Cortical Learning, Neuroscience Research. (2019) 141, 23–28, 10.1016/j.neures.2018.08.004, 2-s2.0-85052064778. 30125609

[90] Goldberg J. S. and Hirschi K. K. , Diverse Roles of the Vasculature Within the Neural Stem Cell Niche, Regenerative Medicine. (2009) 4, no. 6, 879–897, 10.2217/rme.09.61, 2-s2.0-75649144081. 19903006 PMC2836203

[91] Valamparamban G. F. and Spéder P. , Homemade: Building the Structure of the Neurogenic Niche, Frontiers in Cell and Developmental Biology. (2023) 11, 10.3389/fcell.2023.1275963, 1275963. 38107074 PMC10722289

[92] Peguera B. , Segarra M. , and Acker-Palmer A. , Neurovascular Crosstalk Coordinates the Central Nervous System Development, Current Opinion in Neurobiology. (2021) 69, 202–213, 10.1016/j.conb.2021.04.005. 34077852 PMC8411665

[93] Bhattacharya A. , Kaushik D. K. , Lozinski B. M. , and Yong V. W. , Beyond Barrier Functions: Roles of Pericytes in Homeostasis and Regulation of Neuroinflammation, Journal of Neuroscience Research. (2020) 98, no. 12, 2390–2405, 10.1002/jnr.24715. 32815569

[94] Ding R. , Hase Y. , and Ameen-Ali K. E. , et al.Loss of Capillary Pericytes and the Blood–Brain Barrier in White Matter in Poststroke and Vascular Dementias and Alzheimer’s Disease, Brain Pathology. (2020) 30, no. 6, 1087–1101, 10.1111/bpa.12888. 32705757 PMC8018063

[95] Mercier F. , Fractones: Extracellular Matrix Niche Controlling Stem Cell Fate and Growth Factor Activity in the Brain in Health and Disease, Cellular and Molecular Life Sciences. (2016) 73, no. 24, 4661–4674, 10.1007/s00018-016-2314-y, 2-s2.0-84979970406. 27475964 PMC11108427

[96] Nascimento M. A. , Sorokin L. , and Coelho-Sampaio T. , Fractone Bulbs Derive From Ependymal Cells and Their Laminin Composition Influence the Stem Cell Niche in the Subventricular Zone, The Journal of Neuroscience. (2018) 38, no. 16, 3880–3889, 10.1523/JNEUROSCI.3064-17.2018, 2-s2.0-85050949383. 29530987 PMC6705924

[97] Kim H. J. , Lee E. , and Nam M. , et al.Contribution of Extracellular Matrix Component Landscapes in the Adult Subventricular Zone to the Positioning of Neural Stem/Progenitor Cells, Experimental Neurobiology. (2021) 30, no. 4, 275–284, 10.5607/en21012. 34483142 PMC8424380

[98] Kerever A. , Schnack J. , and Vellinga D. , et al.Novel Extracellular Matrix Structures in the Neural Stem Cell Niche Capture the Neurogenic Factor Fibroblast Growth Factor 2 From the Extracellular Milieu, Stem Cells. (2007) 25, no. 9, 2146–2157, 10.1634/stemcells.2007-0082, 2-s2.0-34748926972. 17569787

[99] Sun Y. , Jin K. , and Xie L. , et al.VEGF-Induced Neuroprotection, Neurogenesis, and Angiogenesis After Focal Cerebral Ischemia, Journal of Clinical Investigation. (2003) 111, no. 12, 1843–1851, 10.1172/JCI200317977, 2-s2.0-0041743159. 12813020 PMC161428

[100] Wu S.-Y. , Pan B.-S. , and Tsai S.-F. , et al.BDNF Reverses Aging-Related Microglial Activation, Journal of Neuroinflammation. (2020) 17, no. 1, 10.1186/s12974-020-01887-1, 210. 32664974 PMC7362451

[101] Guo S. , Kim W. J. , and Lok J. , et al.Neuroprotection via Matrix-Trophic Coupling Between Cerebral Endothelial Cells and Neurons, Proceedings of the National Academy of Sciences. (2008) 105, no. 21, 7582–7587, 10.1073/pnas.0801105105, 2-s2.0-44949103395. PMC2396701 18495934

[102] Zamora M. , Clapp C. , de la Escalera G. M. , and Robles J. P. , Integrin α5β1 Mediates the Inhibitory Effects of Vasoinhibin on Angiogenesis and Vascular Permeability, 2025, bioRxiv. 10.1016/j.jbc.2025.110978 PMC12795676 41308993

[103] Chen T. , Illand A. , and Tsai C. , et al.Actin-Driven Nanotopography Enhances Integrin Molecular Clutch in Developing Tissue, 2023.

[104] Gao B. , Zhou S. , and Sun C. , et al.Brain Endothelial Cell-Derived Exosomes Induce Neuroplasticity in Rats With Ischemia/Reperfusion Injury, ACS Chemical Neuroscience. (2020) 11, no. 15, 2201–2213, 10.1021/acschemneuro.0c00089. 32574032

[105] Liu Y. , Luo Z. , and Xie Y. , et al.Extracellular Vesicles From UTX-Knockout Endothelial Cells Boost Neural Stem Cell Differentiation in Spinal Cord Injury, Cell Communication and Signaling. (2024) 22, no. 1, 10.1186/s12964-023-01434-4, 155. 38424563 PMC10903014

[106] Huang J. , Zhang G. , and Li S. , et al.Endothelial Cell-Derived Exosomes Boost and Maintain Repair-Related Phenotypes of Schwann Cells via miR199-5p to Promote Nerve Regeneration, Journal of Nanobiotechnology. (2023) 21, no. 1, 10.1186/s12951-023-01767-9, 10. 36624511 PMC9827708

[107] Lombardo G. , Gili M. , and Grange C. , et al.IL-3R-Alpha Blockade Inhibits Tumor Endothelial Cell-Derived Extracellular Vesicle (EV)-Mediated Vessel Formation by Targeting the β-Catenin Pathway, Oncogene. (2018) 37, no. 9, 1175–1191, 10.1038/s41388-017-0034-x, 2-s2.0-85038009127. 29238040 PMC5861089

[108] Visvanathan J. , Lee S. , Lee B. , Lee J. W. , and Lee S.-K. , The microRNA miR-124 Antagonizes the Anti-Neural REST/SCP1 Pathway During Embryonic CNS Development, Genes & Development. (2007) 21, no. 7, 744–749, 10.1101/gad.1519107, 2-s2.0-34147157651. 17403776 PMC1838526

[109] Cheng L. C. , Pastrana E. , Tavazoie M. , and Doetsch F. , miR-124 Regulates Adult Neurogenesis in the Subventricular Zone Stem Cell Niche, Nature Neuroscience. (2009) 12, no. 4, 399–408, 10.1038/nn.2294, 2-s2.0-63649138643. 19287386 PMC2766245

[110] Zhou F. , Zhang C. , and Guan Y. , et al.Screening the Expression Characteristics of Several miRNAs in G93A-SOD1 Transgenic Mouse: Altered Expression of miRNA-124 Is Associated With Astrocyte Differentiation by Targeting Sox2 and Sox9, Journal of Neurochemistry. (2018) 145, no. 1, 51–67, 10.1111/jnc.14229, 2-s2.0-85045529685. 28960306

[111] Saraiva C. , Paiva J. , Santos T. , Ferreira L. , and Bernardino L. , MicroRNA-124 Loaded Nanoparticles Enhance Brain Repair in Parkinson’s Disease, Journal of Controlled Release. (2016) 235, 291–305, 10.1016/j.jconrel.2016.06.005, 2-s2.0-84974633324. 27269730

[112] Pan Q. , Wang Y. , and Xiang Z. , et al.NSC-Derived Extracellular Vesicles-Mediates Neuronal Plasticity Enhancement in Vascular Dementia via Transferring miR-210, Acta Neuropathologica Communications. (2025) 13, no. 1, 10.1186/s40478-025-02073-1, 152. 40635095 PMC12239277

[113] Karshovska E. , Wei Y. , and Subramanian P. , et al.HIF-1α (Hypoxia-Inducible Factor-1α) Promotes Macrophage Necroptosis by Regulating miR-210 and miR-383, Arteriosclerosis, Thrombosis, and Vascular Biology. (2020) 40, no. 3, 583–596, 10.1161/ATVBAHA.119.313290. 31996026

[114] Rengarajan A. , Goldblatt H. E. , Beebe D. J. , Virumbrales-Muñoz M. , and Boeldt D. S. , Immune Cells and Inflammatory Mediators cause Endothelial Dysfunction in a Vascular Microphysiological System, Lab on a Chip. (2024) 24, no. 6, 1808–1820, 10.1039/D3LC00824J. 38363157 PMC11022267

[115] AlZaim I. , de Rooij L. P. , Sheikh B. N. , Börgeson E. , and Kalucka J. , The Evolving Functions of the Vasculature in Regulating Adipose Tissue Biology in Health and Obesity, Nature Reviews Endocrinology. (2023) 19, no. 12, 691–707, 10.1038/s41574-023-00893-6. 37749386

[116] Sharma K. , Zhang Y. , Paudel K. R. , Kachelmeier A. , Hansbro P. M. , and Shi X. , The Emerging Role of Pericyte-Derived Extracellular Vesicles in Vascular and Neurological Health, Cells. (2022) 11, no. 19, 10.3390/cells11193108, 3108. 36231071 PMC9563036

[117] Zagrean A.-M. , Hermann D. M. , Opris I. , Zagrean L. , and Popa-Wagner A. , Multicellular Crosstalk Between Exosomes and the Neurovascular Unit After Cerebral Ischemia. Therapeutic Implications, Frontiers in Neuroscience. (2018) 12, 10.3389/fnins.2018.00811, 2-s2.0-85057141847, 811. 30459547 PMC6232510

[118] Ruan H. , Li Y. , and Wang C. , et al.Click Chemistry Extracellular Vesicle/Peptide/Chemokine Nanocarriers for Treating Central Nervous System Injuries, Acta Pharmaceutica Sinica B. (2023) 13, no. 5, 2202–2218, 10.1016/j.apsb.2022.06.007. 37250158 PMC10213615

[119] Chen C. , Cai N. , Niu Q. , Tian Y. , Hu Y. , and Yan X. , Quantitative Assessment of Lipophilic Membrane Dye-Based Labelling of Extracellular Vesicles by Nano-Flow Cytometry, Journal of Extracellular Vesicles. (2023) 12, no. 8, 10.1002/jev2.12351, 12351. 37525378 PMC10390660

[120] Haines L. A. , Baeckler A. A. , and Schofield S. J. , et al.Non-Specific Particle Formation During Extracellular Vesicle Labelling With the Lipophilic Membrane Dye PKH26, Journal of Extracellular Vesicles. (2025) 14, no. 5, 10.1002/jev2.70079, e70079. 40387660 PMC12087298

[121] Jensen K. H. and Berg R. W. , CLARITY-Compatible Lipophilic Dyes for Electrode Marking and Neuronal Tracing, Scientific Reports. (2016) 6, no. 1, 10.1038/srep32674, 2-s2.0-84986212693, 32674. 27597115 PMC5011694

[122] Teo W. , Morgan M. , and Stys P. , Quantitation of the Physicochemical Properties of Myelin Using Nile Red Fluorescence Spectroscopy, Journal of Neurochemistry. (2025) 169, no. 1, 10.1111/jnc.16203, e16203. 39152713 PMC11657930

[123] Schneider J. , Pultar M. , and Oesterreicher J. , et al.Cre mRNA Is Not Transferred by EVs from Endothelial and Adipose-Derived Stromal/Stem Cells During Vascular Network Formation, International Journal of Molecular Sciences. (2021) 22, no. 8, 10.3390/ijms22084050, 4050. 33919955 PMC8070972

[124] Clares-Pedrero I. , Rocha-Mulero A. , Palma-Cobo M. , Cardeñes B. , Yáñez-Mó M. , and Cabañas C. , Molecular Determinants Involved in the Docking and Uptake of Tumor-Derived Extracellular Vesicles: Implications in Cancer, International Journal of Molecular Sciences. (2024) 25, no. 6, 10.3390/ijms25063449, 3449. 38542421 PMC10970554

[125] Toribio V. and Yanez-Mo M. , Tetraspanins Interweave EV Secretion, Endosomal Network Dynamics and Cellular Metabolism, European Journal of Cell Biology. (2022) 101, no. 3, 10.1016/j.ejcb.2022.151229, 151229. 35500468

[126] Murphy D. E. , de Jong O. G. , and Brouwer M. , et al.Extracellular Vesicle-Based Therapeutics: Natural Versus Engineered Targeting and Trafficking, Experimental & Molecular Medicine. (2019) 51, no. 3, 1–12, 10.1038/s12276-019-0223-5, 2-s2.0-85063006199. PMC6418170 30872574

[127] Kang M. , Jordan V. , Blenkiron C. , and Chamley L. W. , Biodistribution of Extracellular Vesicles Following Administration Into Animals: A Systematic Review, Journal of Extracellular Vesicles. (2021) 10, no. 8, 10.1002/jev2.12085, e12085. 34194679 PMC8224174

[128] Colombo F. , Nimkar K. , Norton E. G. , Lovat F. , and Cocucci E. , Exploring the Spatial Limits of Extracellular Vesicles-Mediated Intercellular Communication, Journal of Extracellular Vesicles. (2025) 14, no. 11, 10.1002/jev2.70169, e70169. 41167986 PMC12575057

[129] Reymond S. , Vujić T. , and Sanchez J.-C. , Neurovascular Unit-Derived Extracellular Vesicles: From Their Physiopathological Roles to Their Clinical Applications in Acute Brain Injuries, Biomedicines. (2022) 10, no. 9, 10.3390/biomedicines10092147, 2147. 36140248 PMC9495841

[130] Licht T. , Sasson E. , and Bell B. , et al.Hippocampal Neural Stem Cells Facilitate Access From Circulation via Apical Cytoplasmic Processes, eLife. (2020) 9, 10.7554/eLife.52134, e52134. 32496193 PMC7297534

[131] Joshi B. S. and Zuhorn I. S. , Heparan Sulfate Proteoglycan-Mediated Dynamin-Dependent Transport of Neural Stem Cell Exosomes in an In Vitro Blood–brain Barrier Model, European Journal of Neuroscience. (2021) 53, no. 3, 706–719, 10.1111/ejn.14974. 32939863 PMC7891616

[132] Mori T. , Buffo A. , and Götz M. , The Novel Roles of Glial Cells Revisited: The Contribution of Radial Glia and Astrocytes to Neurogenesis, Current Topics in Developmental Biology. (2005) 69, 67–99. 16243597 10.1016/S0070-2153(05)69004-7

[133] Paone L. S. , Benmassaoud M. M. , Curran A. , Vega Sán L. , and Galie P. A. , A 3D-Printed Blood-Brain Barrier Model With Tunable Topology and Cell-Matrix Interactions, Biofabrication. (2023) 16, no. 1, 10.1088/1758-5090/ad0260, 015005. 37820611

[134] Nagata S. and Yamasaki R. , The Involvement of Glial Cells in Blood–Brain Barrier Damage in Neuroimmune Diseases, International Journal of Molecular Sciences. (2024) 25, no. 22, 10.3390/ijms252212323, 12323. 39596390 PMC11594741

[135] Tabata H. , Crosstalk Between Blood Vessels and Glia during the Central Nervous System Development, Life. (2022) 12, no. 11, 10.3390/life12111761, 1761. 36362915 PMC9699316

[136] Segarra M. , Aburto M. R. , and Acker-Palmer A. , Blood–Brain Barrier Dynamics to Maintain Brain Homeostasis, Trends in Neurosciences. (2021) 44, no. 5, 393–405, 10.1016/j.tins.2020.12.002. 33423792

[137] Sofroniew M. V. , Reactive Astrocytes in Neural Repair and Protection, The Neuroscientist. (2005) 11, no. 5, 400–407, 10.1177/1073858405278321, 2-s2.0-25144440471. 16151042

[138] Pekny M. and Pekna M. , Astrocyte Reactivity and Reactive Astrogliosis: Costs and Benefits, Physiological Reviews. (2014) 94, no. 4, 1077–1098, 10.1152/physrev.00041.2013, 2-s2.0-84921791889. 25287860

[139] Clarke B. E. and Patani R. , The Microglial Component of Amyotrophic Lateral Sclerosis, Brain. (2020) 143, no. 12, 3526–3539, 10.1093/brain/awaa309. 33427296 PMC7805793

[140] Young A. M. , Kumasaka N. , and Calvert F. , et al.A Map of Transcriptional Heterogeneity and Regulatory Variation in Human Microglia, Nature Genetics. (2021) 53, no. 6, 861–868, 10.1038/s41588-021-00875-2. 34083789 PMC7610960

[141] Anderson S. R. , Roberts J. M. , and Ghena N. , et al.Neuronal Apoptosis Drives Remodeling States of Microglia and Shifts in Survival Pathway Dependence, eLife. (2022) 11, 10.7554/eLife.76564, e76564. 35481836 PMC9071266

[142] Park J. C. , Han J. W. , and Lee W. , et al.Microglia Gravitate toward Amyloid Plaques Surrounded by Externalized Phosphatidylserine via TREM2, Advanced Science. (2024) 11, no. 34, 10.1002/advs.202400064, 2400064. 38981007 PMC11425970

[143] Zhao P. , Xu Y. , and Jiang L.-L. , et al.LILRB2-Mediated TREM2 Signaling Inhibition Suppresses Microglia Functions, Molecular Neurodegeneration. (2022) 17, no. 1, 10.1186/s13024-022-00550-y, 44. 35717259 PMC9206387

[144] Xie M. , Liu Y. U. , and Zhao S. , et al.TREM2 Interacts With TDP-43 and Mediates Microglial Neuroprotection Against TDP-43-Related Neurodegeneration, Nature Neuroscience. (2022) 25, no. 1, 26–38, 10.1038/s41593-021-00975-6. 34916658 PMC8741737

[145] Jiang X. , Yi S. , Liu Q. , and Zhang J. , The Secretome of Microglia Induced by IL-4 of IFN-γ Differently Regulate Proliferation, Differentiation and Survival of Adult Neural Stem/Progenitor Cell by Targeting the PI3K-Akt Pathway, Cytotechnology. (2022) 74, no. 3, 407–420, 10.1007/s10616-022-00534-2. 35733698 PMC9206954

[146] Villa A. , Gelosa P. , and Castiglioni L. , et al.Sex-Specific Features of Microglia from Adult Mice, Cell Reports. (2018) 23, no. 12, 3501–3511, 10.1016/j.celrep.2018.05.048, 2-s2.0-85048555873. 29924994 PMC6024879

[147] Nelson L. H. , Peketi P. , and Lenz K. M. , Microglia Regulate Cell Genesis in a Sex-dependent Manner in the Neonatal Hippocampus, Neuroscience. (2021) 453, 237–255, 10.1016/j.neuroscience.2020.10.009. 33129890

[148] Yanguas-Casás N. , Physiological Sex Differences in Microglia and Their Relevance in Neurological Disorders, Neuroimmunology and Neuroinflammation. (2020) 7, no. 1, 13–22, 10.20517/2347-8659.2019.31.

[149] between Cellular Growth and Atypical Fusion Defines Morphogenesis of a Modular Glial Niche in Drosophila, Nature Communications. (2022) 13, no. 1, 10.1038/s41467-022-32685-3, 4999. PMC9411534 36008397

[150] Machado L. S. , Ferreira P. S. , and Pires M. R. , et al.3D Bioprinted Human iPSC-Derived Neural Progenitor Cells as a Novel Platform for Studying Neurogenic Niche, APL Bioengineering. (2025) 9, no. 3, 10.1063/5.0276704. PMC12422755 40936945

[151] Vicidomini C. , Guo N. , and Sahay A. , Communication, Cross Talk, and Signal Integration in the Adult Hippocampal Neurogenic Niche, Neuron. (2020) 105, no. 2, 220–235, 10.1016/j.neuron.2019.11.029. 31972145 PMC7184932

[152] Bjornsson C. S. , Apostolopoulou M. , Tian Y. , and Temple S. , It Takes a Village: Constructing the Neurogenic Niche, Developmental Cell. (2015) 32, no. 4, 435–446, 10.1016/j.devcel.2015.01.010, 2-s2.0-84923241642. 25710530 PMC4886554

[153] Dulken B. W. , Buckley M. T. , and Navarro Negredo P. , et al.Single-Cell Analysis Reveals T Cell Infiltration in Old Neurogenic Niches, Nature. (2019) 571, no. 7764, 205–210, 10.1038/s41586-019-1362-5, 2-s2.0-85068836797. 31270459 PMC7111535

[154] Matejuk A. and Ransohoff R. M. , Crosstalk Between Astrocytes and Microglia: An Overview, Frontiers in Immunology. (2020) 11, 10.3389/fimmu.2020.01416, 1416. 32765501 PMC7378357

[155] Vainchtein I. D. and Molofsky A. V. , Astrocytes and Microglia: In Sickness and in Health, Trends in Neurosciences. (2020) 43, no. 3, 144–154, 10.1016/j.tins.2020.01.003. 32044129 PMC7472912

[156] Faust T. E. , Lee Y.-H. , and O’Connor C. D. , et al.Microglia-Astrocyte Crosstalk Regulates Synapse Remodeling via Wnt Signaling, Cell. (2025) 188, no. 19, 5212–5230.e21, 10.1016/j.cell.2025.08.023. 40934914 PMC12489809

[157] Zhu H. , Cao Y. , and Chen X. , et al.CX3CL1 Attenuates Neurological Deficit and Neuroinflammation Through CX3CR1/p38 MAPK/ERK1/2 Signaling Pathway in Traumatic Brain Injury, International Immunopharmacology. (2026) 168, 10.1016/j.intimp.2025.115765, 115765. 41192110

[158] Araki T. , Ikegaya Y. , and Koyama R. , The Effects of Microglia-and Astrocyte-Derived Factors on Neurogenesis in Health and Disease, European Journal of Neuroscience. (2021) 54, no. 5, 5880–5901, 10.1111/ejn.14969. 32920880 PMC8451940

[159] Liu S. and Zhou S. , Lactate: A New Target for Brain Disorders, Neuroscience. (2024) 552, 100–111, 10.1016/j.neuroscience.2024.06.023. 38936457

[160] Ishijima T. and Nakajima K. , Inflammatory Cytokines TNFα, IL-1β, and IL-6 Are Induced in Endotoxin-Stimulated Microglia Through Different Signaling Cascades, Science Progress. (2021) 104, no. 4, 10.1177/00368504211054985, 00368504211054985. 34821182 PMC10450609

[161] Tujula I. , Hyvärinen T. , and Lotila J. , et al.Modeling Neuroinflammatory Interactions Between Microglia and Astrocytes in a Human iPSC-Based Coculture Platform, Cell Communication and Signaling. (2025) 23, no. 1, 10.1186/s12964-025-02304-x, 298. 40542355 PMC12181861

[162] Wang Y. , Li J. , Wang M.-Y. , Pan Z.-Y. , Li Z.-Q. , and Wang Z.-F. , Chronic Microglial Inflammation Promotes Neuronal Lactate Supply but Impairs Its Utilization in Primary Rat Astrocyte-Neuron Co-Cultures, Biochemical and Biophysical Research Communications. (2022) 607, 28–35, 10.1016/j.bbrc.2022.03.122. 35366540

[163] Trindade A. and Duarte A. , Notch Signaling Function in the Angiocrine Regulation of Tumor Development, Cells. (2020) 9, no. 11, 10.3390/cells9112467, 2467. 33198378 PMC7697556

[164] Li Q. , Ford M. C. , Lavik E. B. , and Madri J. A. , Modeling the Neurovascular Niche: VEGF-and BDNF-Mediated Cross-Talk Between Neural Stem Cells and Endothelial Cells: An In Vitro Study, Journal of Neuroscience Research. (2006) 84, no. 8, 1656–1668, 10.1002/jnr.21087, 2-s2.0-33845383924. 17061253

[165] Guérit S. , Fidan E. , and Macas J. , et al.Astrocyte-Derived Wnt Growth Factors Are Required for Endothelial Blood-Brain Barrier Maintenance, Progress in Neurobiology. (2021) 199, 10.1016/j.pneurobio.2020.101937, 101937. 33383106

[166] Czpakowska J. , Głąbiński A. , and Szpakowski P. , The Potential Role of Exosomes in Communication Between Astrocytes and Endothelial Cells, International Journal of Molecular Sciences. (2025) 26, no. 10, 10.3390/ijms26104676, 4676. 40429819 PMC12111803

[167] Amersfoort J. , Eelen G. , and Carmeliet P. , Immunomodulation by Endothelial Cells—Partnering Up With the Immune System?, Nature Reviews Immunology. (2022) 22, no. 9, 576–588, 10.1038/s41577-022-00694-4. PMC8920067 35288707

[168] Greene C. , Hanley N. , and Campbell M. , Claudin-5: Gatekeeper of Neurological Function, Fluids and Barriers of the CNS. (2019) 16, no. 1, 10.1186/s12987-019-0123-z, 2-s2.0-85060568804, 3. 30691500 PMC6350359

[169] Selim M. S. , Matani B. R. , Henry-Ojo H. O. , Narayanan S. P. , and Somanath P. R. , Claudin 5 Across the Vascular Landscape: From Blood–Tissue Barrier Regulation to Disease Mechanisms, Cells. (2025) 14, no. 17, 10.3390/cells14171346, 1346. 40940757 PMC12427713

[170] Chen A.-Q. , Fang Z. , and Chen X.-L. , et al.Microglia-Derived TNF-α Mediates Endothelial Necroptosis Aggravating Blood Brain–Barrier Disruption After Ischemic Stroke, Cell Death & Disease. (2019) 10, no. 7, 10.1038/s41419-019-1716-9, 2-s2.0-85067619814, 487. 31221990 PMC6586814

[171] Jiao H. , Wang Z. , Liu Y. , Wang P. , and Xue Y. , Specific Role of Tight Junction Proteins Claudin-5, Occludin, and ZO-1 of the Blood–Brain Barrier in a Focal Cerebral Ischemic Insult, Journal of Molecular Neuroscience. (2011) 44, no. 2, 130–139, 10.1007/s12031-011-9496-4, 2-s2.0-79955922988. 21318404

[172] Simpson D. S. and Oliver P. L. , ROS Generation in Microglia: Understanding Oxidative Stress and Inflammation in Neurodegenerative Disease, Antioxidants. (2020) 9, no. 8, 10.3390/antiox9080743, 743. 32823544 PMC7463655

[173] Dupret D. , Fabre A. , and Döbrössy M. D. , et al.Spatial Learning Depends on Both the Addition and Removal of New Hippocampal Neurons, PLoS Biology. (2007) 5, no. 8, 10.1371/journal.pbio.0050214, 2-s2.0-34548249099, e214. 17683201 PMC1939885

[174] Tronel S. , Fabre A. , Charrier V. , Oliet S. H. , Gage F. H. , and Abrous D. N. , Spatial Learning Sculpts the Dendritic Arbor of Adult-Born Hippocampal Neurons, Proceedings of the National Academy of Sciences. (2010) 107, no. 17, 7963–7968, 10.1073/pnas.0914613107, 2-s2.0-77952382816. PMC2867872 20375283

[175] Kirshenbaum G. S. , Chang C.-Y. , and Bompolaki M. , et al.Adult-Born Neurons Maintain Hippocampal Cholinergic Inputs and Support Working Memory During Aging, Molecular Psychiatry. (2023) 28, no. 12, 5337–5349, 10.1038/s41380-023-02167-z. 37479778 PMC12958120

[176] Hammond T. R. , Dufort C. , and Dissing-Olesen L. , et al.Single-Cell RNA Sequencing of Microglia throughout the Mouse Lifespan and in the Injured Brain Reveals Complex Cell-State Changes, Immunity. (2019) 50, no. 1, 253–271.e6, 10.1016/j.immuni.2018.11.004, 2-s2.0-85056738677. 30471926 PMC6655561

[177] Wang J. , He W. , and Zhang J. , A Richer and More Diverse Future for Microglia Phenotypes, Heliyon. (2023) 9, no. 4, 10.1016/j.heliyon.2023.e14713, e14713. 37025898 PMC10070543

[178] Cadiz M. P. , Jensen T. D. , and Sens J. P. , et al.Culture Shock: Microglial Heterogeneity, Activation, and Disrupted Single-Cell Microglial Networks In Vitro, Molecular Neurodegeneration. (2022) 17, no. 1, 10.1186/s13024-022-00531-1, 26. 35346293 PMC8962153

[179] Pettas S. , Karagianni K. , and Kanata E. , et al.Profiling Microglia Through Single-Cell RNA Sequencing Over the Course of Development, Aging, and Disease, Cells. (2022) 11, no. 15, 10.3390/cells11152383, 2383. 35954228 PMC9368511

[180] Ochocka N. , Segit P. , and Walentynowicz K. A. , et al.Single-Cell RNA Sequencing Reveals Functional Heterogeneity of Glioma-Associated Brain Macrophages, Nature Communications. (2021) 12, no. 1, 10.1038/s41467-021-21407-w, 1151. PMC7895824 33608526

[181] Deczkowska A. , Keren-Shaul H. , Weiner A. , Colonna M. , Schwartz M. , and Amit I. , Disease-Associated Microglia: A Universal Immune Sensor of Neurodegeneration, Cell. (2018) 173, no. 5, 1073–1081, 10.1016/j.cell.2018.05.003, 2-s2.0-85046853468. 29775591

[182] Keren-Shaul H. , Spinrad A. , and Weiner A. , et al.A Unique Microglia Type Associated With Restricting Development of Alzheimer’s Disease, Cell. (2017) 169, no. 7, 1276–1290.e17, 10.1016/j.cell.2017.05.018, 2-s2.0-85020287843. 28602351

[183] Labuda V. and Sundara M. A. , The Role of Disease-Associated Microglia in Neurodegenerative Disease: A Review, Undergraduate Research in Natural and Clinical Science and Technology Journal. (2024) 8, 1–9, 10.26685/urncst.575.

[184] Pitts K. M. and Margeta M. A. , Myeloid Masquerade: Microglial Transcriptional Signatures in Retinal Development and Disease, Frontiers in Cellular Neuroscience. (2023) 17, 10.3389/fncel.2023.1106547, 1106547. 36779012 PMC9909491

[185] Atagi Y. , Liu C.-C. , and Painter M. M. , et al.Apolipoprotein E Is a Ligand for Triggering Receptor Expressed on Myeloid Cells 2 (TREM2), Journal of Biological Chemistry. (2015) 290, no. 43, 26043–26050, 10.1074/jbc.M115.679043, 2-s2.0-84944937240. 26374899 PMC4646257

[186] Jendresen C. , Årskog V. , Daws M. R. , and Nilsson L. N. , The Alzheimer’s Disease Risk Factors Apolipoprotein E and TREM2 Are Linked in a Receptor Signaling Pathway, Journal of Neuroinflammation. (2017) 14, no. 1, 10.1186/s12974-017-0835-4, 2-s2.0-85015758641, 59. 28320424 PMC5360024

[187] Clemenson G. D. , Deng W. , and Gage F. H. , Environmental Enrichment and Neurogenesis: From Mice to Humans, Current Opinion in Behavioral Sciences. (2015) 4, 56–62, 10.1016/j.cobeha.2015.02.005, 2-s2.0-84924747728.

[188] Steventon J. , Foster C. , Furby H. , Helme D. , Wise R. , and Murphy K. , Hippocampal Blood Flow Is Increased After 20 Min of Moderate-Intensity Exercise, Cerebral Cortex. (2020) 30, no. 2, 525–533, 10.1093/cercor/bhz104. 31216005 PMC7703728

[189] Walsh H. J. , Saito S. , Kunimatsu N. , Karaki M. , Fisher J. P. , and Ogoh S. , Effects of Interval Versus Continuous Exercise on Cerebral Vascular Flow-Mediated Dilatation in Young Healthy Males, Physiological Reports. (2025) 13, no. 9, 10.14814/phy2.70354, e70354. 40323188 PMC12051378

[190] Sakamoto R. , Kamoda T. , and Sato K. , et al.Acute Aerobic Exercise Enhances Cerebrovascular Shear-Mediated Dilation in Young Adults: The Role of Cerebral Shear, Journal of Applied Physiology. (2024) 136, no. 3, 535–548, 10.1152/japplphysiol.00543.2023. 38153849

[191] Yuan T.-F. , Barbosa Ferreira Rocha N. , Paes F. , Arias-Carrión O. , Machado S. , and Souza de Sá Filho A. , Neural Mechanism of Exercise: Neurovascular Responses to Exercise, CNS & Neurological Disorders - Drug Targets. (2015) 14, no. 10, 1304–1306, 10.2174/1871527315666151111124543, 2-s2.0-84959518063. 26556076

[192] Han J. , Calvo C.-F. , and Kang T. H. , et al.Vascular Endothelial Growth Factor Receptor 3 Controls Neural Stem Cell Activation in Mice and Humans, Cell Reports. (2015) 10, no. 7, 1158–1172, 10.1016/j.celrep.2015.01.049, 2-s2.0-84924530916. 25704818 PMC4685253

[193] Matta R. , Feng Y. , Sansing L. H. , and Gonzalez A. L. , Endothelial Cell Secreted VEGF-C Enhances NSC VEGFR3 Expression and Promotes NSC Survival, Stem Cell Research. (2021) 53, 10.1016/j.scr.2021.102318, 102318. 33836422 PMC8243729

[194] Jiang H. , Kimura Y. , and Inoue S. , et al.Effects of Different Exercise Modes and Intensities on Cognitive Performance, Adult Hippocampal Neurogenesis, and Synaptic Plasticity in Mice, Experimental Brain Research. (2024) 242, no. 7, 1709–1719, 10.1007/s00221-024-06854-3. 38806710

[195] Nokia M. S. , Lensu S. , and Ahtiainen J. P. , et al.Physical Exercise Increases Adult Hippocampal Neurogenesis in Male Rats provided It Is Aerobic and Sustained, The Journal of Physiology. (2016) 594, no. 7, 1855–1873, 10.1113/JP271552. 26844666 PMC4818598

[196] Ben-Zeev T. , Shoenfeld Y. , and Hoffman J. R. , The Effect of Exercise on Neurogenesis in the Brain, The Israel Medical Association Journal: IMAJ. (2022) 24, no. 8, 533–538. 35971998

[197] Osadchiy V. , Martin C. R. , and Mayer E. A. , The Gut–Brain Axis and the Microbiome: Mechanisms and Clinical Implications, Clinical Gastroenterology and Hepatology. (2019) 17, no. 2, 322–332, 10.1016/j.cgh.2018.10.002. 30292888 PMC6999848

[198] Scott G. A. , Terstege D. J. , Vu A. P. , Law S. , Evans A. , and Epp J. R. , Disrupted Neurogenesis in Germ-Free Mice: Effects of Age and Sex, Frontiers in Cell and Developmental Biology. (2020) 8, 10.3389/fcell.2020.00407, 407. 32548122 PMC7272680

[199] Post Z. , Manfready R. A. , and Keshavarzian A. , Overview of the Gut–Brain Axis: From Gut to Brain and Back Again, Seminars in Neurology. (2023) 43, no. 4, 506–517. 37562457 10.1055/s-0043-1771464

[200] Chevalier G. , Siopi E. , and Guenin-Macé L. , et al.Effect of Gut Microbiota on Depressive-Like Behaviors in Mice Is Mediated by the Endocannabinoid System, Nature Communications. (2020) 11, no. 1, 10.1038/s41467-020-19931-2, 6363. PMC7732982 33311466

[201] Del Toro-Barbosa M. , Hurtado-Romero A. , Garcia-Amezquita L. E. , and García-Cayuela T. , Psychobiotics: Mechanisms of Action, Evaluation Methods and Effectiveness in Applications With Food Products, Nutrients. (2020) 12, no. 12, 10.3390/nu12123896, 3896. 33352789 PMC7767237

[202] Misera A. , Liśkiewicz P. , Łoniewski I. , Skonieczna-Żydecka K. , and Samochowiec J. , Effect of Psychobiotics on Psychometric Tests and Inflammatory Markers in Major Depressive Disorder: Meta-Analysis of Randomized Controlled Trials With Meta-Regression, Pharmaceuticals. (2021) 14, no. 10, 10.3390/ph14100952, 952. 34681176 PMC8541446

[203] Medawar E. , Beyer F. , and Thieleking R. , et al.Prebiotic Diet Changes Neural Correlates of Food Decision-Making in Overweight Adults: A Randomised Controlled Within-Subject Cross-Over Trial, Gut. (2024) 73, no. 2, 298–310, 10.1136/gutjnl-2023-330365. 37793780 PMC10850731

[204] Bonaz B. , Bazin T. , and Pellissier S. , The Vagus Nerve at the Interface of the Microbiota-Gut-Brain Axis, Frontiers in Neuroscience. (2018) 12, 10.3389/fnins.2018.00049, 336468. PMC5808284 29467611

[205] Forsythe P. , Bienenstock J. , and Kunze W. A. , Vagal Pathways for Microbiome-Brain-Gut Axis Communication, Advances in Experimental Medicine and Biology, 2014, 817, 115–133. 10.1007/978-1-4939-0897-4_5 24997031

[206] Suarez A. N. , Hsu T. M. , and Liu C. M. , et al.Gut Vagal Sensory Signaling Regulates Hippocampus Function Through Multi-Order Pathways, Nature Communications. (2018) 9, no. 1, 10.1038/s41467-018-04639-1, 2181. PMC5988686 29872139

[207] O’Leary O. F. , Ogbonnaya E. S. , and Felice D. , et al.The Vagus Nerve Modulates BDNF Expression and Neurogenesis in the Hippocampus, European Neuropsychopharmacology. (2018) 28, no. 2, 307–316, 10.1016/j.euroneuro.2017.12.004. 29426666

[208] Dai Y. , Liu S. , Chen J. , Liu L. , Zhou C. , and Zuo Y. , Microglial Responses and Pain Behaviors Are Exacerbated by Chronic Sleep Deprivation in Rats With Chronic Pain Via Neuroinflammatory Pathways, Neuroscience. (2022) 503, 83–94, 10.1016/j.neuroscience.2022.09.004. 36096338

[209] Lazarov O. , Robinson J. , and Tang Y.-P. , et al.Environmental Enrichment Reduces Aβ Levels and Amyloid Deposition in Transgenic Mice, Cell. (2005) 120, no. 5, 701–713, 10.1016/j.cell.2005.01.015. 15766532

[210] Baroncelli L. , Braschi C. , Spolidoro M. , Begenisic T. , Sale A. , and Maffei L. , Nurturing Brain Plasticity: Impact of Environmental Enrichment, Cell Death & Differentiation. (2010) 17, no. 7, 1092–1103, 10.1038/cdd.2009.193. 20019745

[211] Martikainen M.-V. , Aakko-Saksa P. , and Van Den Broek L. , et al.TUBE Project: Transport-Derived Ultrafines and the Brain Effects, International Journal of Environmental Research and Public Health. (2022) 19, no. 1, 10.3390/ijerph19010311, 311. PMC8751045 35010571

[212] Woodward N. , Haghani A. , and Johnson R. , et al.Prenatal and Early Life Exposure to Air Pollution Induced Hippocampal Vascular Leakage and Impaired Neurogenesis in Association With Behavioral Deficits, Translational Psychiatry. (2018) 8, no. 1, 10.1038/s41398-018-0317-1, 261. 30498214 PMC6265287

[213] Olsson T. , Barcellos L. F. , and Alfredsson L. , Interactions Between Genetic, Lifestyle and Environmental Risk Factors for Multiple Sclerosis, Nature Reviews Neurology. (2017) 13, no. 1, 25–36, 10.1038/nrneurol.2016.187. 27934854

[214] Bravo J. A. , Forsythe P. , and Chew M. V. , et al.Ingestion of Lactobacillus Strain Regulates Emotional Behavior and Central GABA Receptor Expression in a Mouse via the Vagus Nerve, Proceedings of the National Academy of Sciences of the United States of America. (2011) 108, no. 38, 16050–16055, 10.1073/pnas.1102999108. 21876150 PMC3179073

[215] Tang C.-F. , Wang C.-Y. , and Wang J.-H. , et al.Short-Chain Fatty Acids Ameliorate Depressive-like Behaviors of High Fructose-Fed Mice by Rescuing Hippocampal Neurogenesis Decline and Blood–Brain Barrier Damage, Nutrients. (2022) 14, no. 9, 10.3390/nu14091882, 1882. 35565849 PMC9105414

[216] Mandal G. , Alboni S. , and Cattane N. , et al.The Dietary Ligands, Omega-3 Fatty Acid Endocannabinoids and Short-Chain Fatty Acids Prevent Cytokine-Induced Reduction of Human Hippocampal Neurogenesis and Alter the Expression of Genes Involved in Neuroinflammation and Neuroplasticity, Molecular Psychiatry. (2025) 30, no. 11, 5338–5355, 10.1038/s41380-025-03119-5. 40670679 PMC12532594

[217] Sarkar A. , Lehto S. M. , Harty S. , Dinan T. G. , Cryan J. F. , and Burnet P. W. , Psychobiotics and the Manipulation of Bacteria–Gut–Brain Signals, Trends in Neurosciences. (2016) 39, no. 11, 763–781, 10.1016/j.tins.2016.09.002. 27793434 PMC5102282

[218] Kelly J. R. , Allen A. P. , and Temko A. , et al.Lost in Translation? the Potential Psychobiotic *Lactobacillus rhamnosus* (JB-1) Fails to Modulate Stress or Cognitive Performance in Healthy Male Subjects, Brain, Behavior, and Immunity. (2017) 61, 50–59, 10.1016/j.bbi.2016.11.018. 27865949

[219] Asad A. , Kirk M. , Zhu S. , Dong X. , and Gao M. , Effects of Prebiotics and Probiotics on Symptoms of Depression and Anxiety in Clinically Diagnosed Samples: Systematic Review and Meta-analysis of Randomized Controlled Trials, Nutrition Reviews. (2025) 83, no. 7, e1504–e1520, 10.1093/nutrit/nuae177. 39731509 PMC12166186

[220] Garzone S. , Charitos I. A. , and Mandorino M. , et al.Can We Modulate Our Second Brain and Its Metabolites to Change Our Mood? A Systematic Review on Efficacy, Mechanisms, and Future Directions of “Psychobiotics, International Journal of Molecular Sciences. (2025) 26, no. 5, 10.3390/ijms26051972, 1972. 40076598 PMC11899754

[221] Tzikos G. , Chamalidou E. , and Christopoulou D. , et al.Psychobiotics Ameliorate Depression and Anxiety Status in Surgical Oncology Patients: Results From the ProDeCa Study, Nutrients. (2025) 17, no. 5, 10.3390/nu17050857, 857. 40077722 PMC11901992

[222] Slykerman R. F. , Davies N. , and Vlckova K. , et al.Precision Psychobiotics for Gut–Brain Axis Health: Advancing the Discovery Pipelines to Deliver Mechanistic Pathways and Proven Health Efficacy, Microbial Biotechnology. (2025) 18, no. 1, 10.1111/1751-7915.70079, e70079. 39815671 PMC11735468

[223] Lucassen P. J. , Toni N. , Kempermann G. , Frisen J. , Gage F. H. , and Swaab D. F. , Limits to Human Neurogenesis—Really?, Molecular Psychiatry. (2020) 25, no. 10, 2207–2209, 10.1038/s41380-018-0337-5. 30617274 PMC7515796

[224] Ni J.-J. , Xu Q. , and Yan S.-S. , et al.Gut Microbiota and Psychiatric Disorders: A Two-Sample Mendelian Randomization Study, Frontiers in Microbiology. (2022) 12, 10.3389/fmicb.2021.737197, 737197. 35185808 PMC8856606

[225] Zang Y. , Lai X. , Li C. , Ding D. , Wang Y. , and Zhu Y. , The Role of Gut Microbiota in Various Neurological and Psychiatric Disorders—An Evidence Mapping Based on Quantified Evidence, Mediators of Inflammation. (2023) 2023, no. 1, 10.1155/2023/5127157, 5127157. 36816743 PMC9936509

[226] Lai H. M. , Liu A. K. L. , and Ng H. H. M. , et al.Next Generation Histology Methods for Three-Dimensional Imaging of Fresh and Archival Human Brain Tissues, Nature Communications. (2018) 9, no. 1, 10.1038/s41467-018-03359-w, 1066. PMC5852003 29540691

[227] Akassoglou K. , Davalos D. , and Mendiola A. S. , et al.Pioneering Discovery and Therapeutics at the Brain-Vascular-Immune Interface, Cell. (2024) 187, no. 21, 5871–5876, 10.1016/j.cell.2024.09.018. 39423805 PMC13046387

[228] Woodburn S. C. , Bollinger J. L. , and Wohleb E. S. , The Semantics of Microglia Activation: Neuroinflammation, Homeostasis, and Stress, Journal of Neuroinflammation. (2021) 18, no. 1, 10.1186/s12974-021-02309-6, 258. 34742308 PMC8571840

[229] Kohler S. J. , Williams N. I. , Stanton G. B. , Cameron J. L. , and Greenough W. T. , Maturation Time of New Granule Cells in the Dentate Gyrus of Adult Macaque Monkeys Exceeds 6 Months, Proceedings of the National Academy of Sciences. (2011) 108, no. 25, 10326–10331, 10.1073/pnas.1017099108. PMC3121825 21646517

[230] Sorrells S. F. , Paredes M. F. , and Velmeshev D. , et al.Immature Excitatory Neurons Develop During Adolescence in the Human Amygdala, Nature Communications. (2019) 10, no. 1, 10.1038/s41467-019-10765-1, 2748. PMC6588589 31227709

[231] Akter M. , Kaneko N. , and Herranz-Pérez V. , et al.Dynamic Changes in the Neurogenic Potential in the Ventricular–Subventricular Zone of Common Marmoset During Postnatal Brain Development, Cerebral Cortex. (2020) 30, no. 7, 4092–4109, 10.1093/cercor/bhaa031. 32108222

[232] Snyder J. S. , Recalibrating the Relevance of Adult Neurogenesis, Trends in Neurosciences. (2019) 42, no. 3, 164–178, 10.1016/j.tins.2018.12.001. 30686490

[233] Berdugo-Vega G. , Arias-Gil G. , and López-Fernández A. , et al.Increasing Neurogenesis Refines Hippocampal Activity Rejuvenating Navigational Learning Strategies and Contextual Memory Throughout Life, Nature Communications. (2020) 11, no. 1, 10.1038/s41467-019-14026-z, 135. PMC6952376 31919362

[234] Bakken T. E. , Miller J. A. , and Ding S.-L. , et al.A Comprehensive Transcriptional Map of Primate Brain Development, Nature. (2016) 535, no. 7612, 367–375, 10.1038/nature18637. 27409810 PMC5325728

[235] Sorrells S. F. , Paredes M. F. , and Zhang Z. , et al.Positive Controls in Adults and Children Support That Very Few, If Any, New Neurons Are Born in the Adult Human Hippocampus, The Journal of Neuroscience. (2021) 41, no. 12, 2554–2565, 10.1523/JNEUROSCI.0676-20.2020. 33762407 PMC8018729

[236] Yamashita S. , Antigen Retrieval for Light and Electron Microscopy, Immunohistochem Ageless Biotechnol. (2020) 10, 10.5772/intechopen.80837.

[237] Flor-Garcia M. , Terreros-Roncal J. , Moreno-Jimenez E. P. , Avila J. , Rabano A. , and Llorens-Martin M. , Unraveling Human Adult Hippocampal Neurogenesis, Nature Protocols. (2020) 15, no. 2, 668–693, 10.1038/s41596-019-0267-y. 31915385

[238] Hyoto M. , Imai H. , and Nishida A. , et al.Improvement and Development of Immersion Fixatives for Enhanced Tissue Preservation in the Whole Eyeball Specimens, Journal of Veterinary Medical Science. (2025) 87, no. 12, 1432–1440, 10.1292/jvms.25-0411. 41125395 PMC12712169

[239] Montisci M. , Perilli M. , Gastaldi M. , Kondo T. , and Cecchi R. , Formalin: A Turning Point in Forensic Tissue Preservation, Forensic Science International. (2025) 377, 10.1016/j.forsciint.2025.112654, 112654. 40966927

[240] Gallardo-Caballero M. , Rodríguez-Moreno C. B. , and Álvarez-Méndez L. , et al.Prolonged Fixation and Post-Mortem Delay Impede the Study of Adult Neurogenesis in Mice, Communications Biology. (2023) 6, no. 1, 10.1038/s42003-023-05367-z, 978. 37741930 PMC10517969

[241] Singh Kushwaha S. , Patro N. , and Kumar Patro I. , A Sequential Study of Age-Related Lipofuscin Accumulation in Hippocampus and Striate Cortex of Rats, Annals of Neurosciences. (2019) 25, no. 4, 223–233, 10.1159/000490908. PMC6470335 31000961

[242] Moreno-García A. , Kun A. , Calero O. , Medina M. , and Calero M. , An Overview of the Role of Lipofuscin in Age-Related Neurodegeneration, Frontiers in Neuroscience. (2018) 12, 10.3389/fnins.2018.00464, 464. 30026686 PMC6041410

[243] Sakr N. , Glazova O. , and Shevkova L. , et al.Characterizing and Quenching Autofluorescence in Fixed Mouse Adrenal Cortex Tissue, International Journal of Molecular Sciences. (2023) 24, no. 4, 10.3390/ijms24043432, 3432. 36834842 PMC9968082

[244] Sapio M. R. , King D. M. , and Maric D. , et al.Efficient Removal of Naturally-Occurring Lipofuscin Autofluorescence in Human Nervous Tissue Using High-Intensity White Light, The Journal of Pain. (2025) 30, 10.1016/j.jpain.2025.105359, 105359. 40057214 PMC12074538

[245] Ertürk A. , Deep 3D Histology Powered by Tissue Clearing, Omics and AI, Nature Methods. (2024) 21, no. 7, 1153–1165, 10.1038/s41592-024-02327-1. 38997593

[246] Eraslan G. , Drokhlyansky E. , and Anand S. , et al.Single-Nucleus Cross-Tissue Molecular Reference Maps Toward Understanding Disease Gene Function, Science. (2022) 376, no. 6594, 10.1126/science.abl4290, eabl4290. 35549429 PMC9383269

[247] Dumitru I. , Paterlini M. , and Zamboni M. , et al.Identification of Proliferating Neural Progenitors in the Adult Human Hippocampus, Science. (2025) 389, no. 6755, 58–63, 10.1126/science.adu9575. 40608919

[248] Zhou Y. , Su Y. , and Li S. , et al.Molecular Landscapes of Human Hippocampal Immature Neurons Across Lifespan, Nature. (2022) 607, no. 7919, 527–533, 10.1038/s41586-022-04912-w. 35794479 PMC9316413

[249] Ciceri G. , Baggiolini A. , and Cho H. S. , et al.An Epigenetic Barrier Sets the Timing of Human Neuronal Maturation, Nature. (2024) 626, no. 8000, 881–890, 10.1038/s41586-023-06984-8. 38297124 PMC10881400

[250] Maynard K. R. , Collado-Torres L. , and Weber L. M. , et al.Transcriptome-Scale Spatial Gene Expression in the Human Dorsolateral Prefrontal Cortex, Nature Neuroscience. (2021) 24, no. 3, 425–436, 10.1038/s41593-020-00787-0. 33558695 PMC8095368

[251] Piwecka M. , Rajewsky N. , and Rybak-Wolf A. , Single-Cell and Spatial Transcriptomics: Deciphering Brain Complexity in Health and Disease, Nature Reviews Neurology. (2023) 19, no. 6, 346–362, 10.1038/s41582-023-00809-y. 37198436 PMC10191412

[252] Wang Q. , Zhu H. , and Deng L. , et al.Spatial Transcriptomics: Biotechnologies, Computational Tools, and Neuroscience Applications, Small Methods. (2025) 9, no. 5, 10.1002/smtd.202401107, 2401107. 39760243

[253] Reuss A. M. , Rupprecht P. , and Groos D. , et al.ADISCO: A Broadly Applicable Method for 3D Microscopy of Archival Paraffin-Embedded Human Tissues, 2025, Biorxiv.

[254] Mai H. , Rong Z. , and Zhao S. , et al.Scalable Tissue Labeling and Clearing of Intact Human Organs, Nature Protocols. (2022) 17, no. 10, 2188–2215, 10.1038/s41596-022-00712-8. 35859136

[255] Sun H. , Lai H. M. , and Wu W. , Three-Dimensional Visualization and Analysis of Dendritic Spines in Human Brain Tissue, BioTechniques. (2024) 76, no. 1, 37–42, 10.2144/btn-2023-0078. 37994419

[256] Kempermann G. , Gage F. H. , and Aigner L. , et al.Human Adult Neurogenesis: Evidence and Remaining Questions, Cell Stem Cell. (2018) 23, no. 1, 25–30, 10.1016/j.stem.2018.04.004. 29681514 PMC6035081

[257] Lucassen P. J. , Fitzsimons C. P. , Salta E. , and Maletic-Savatic M. , Adult Neurogenesis, Human After All (again): Classic, Optimized, and Future Approaches, Behavioural Brain Research. (2020) 381, 10.1016/j.bbr.2019.112458, 112458. 31899214

[258] Moreno-Jiménez E. P. , Flor-García M. , and Terreros-Roncal J. , et al.Adult Hippocampal Neurogenesis Is Abundant in Neurologically Healthy Subjects and Drops Sharply in Patients With Alzheimer’s Disease, Nature Medicine. (2019) 25, no. 4, 554–560, 10.1038/s41591-019-0375-9. 30911133

[259] Boldrini M. , Fulmore C. A. , and Tartt A. N. , et al.Human Hippocampal Neurogenesis Persists Throughout Aging, Cell Stem Cell. (2018) 22, no. 4, 589–599.e5, 10.1016/j.stem.2018.03.015. 29625071 PMC5957089

[260] Terreros-Roncal J. , Flor-García M. , and Moreno-Jiménez E. P. , et al.Methods to Study Adult Hippocampal Neurogenesis in Humans and Across the Phylogeny, Hippocampus. (2023) 33, no. 4, 271–306, 10.1002/hipo.23474. 36259116 PMC7614361

[261] Stillman J. M. , Mendes Lopes F. , Lin J.-P. , Hu K. , Reich D. S. , and Schafer D. P. , Lipofuscin-Like Autofluorescence Within Microglia and Its Impact on Studying Microglial Engulfment, Nature Communications. (2023) 14, no. 1, 10.1038/s41467-023-42809-y, 7060. PMC10624656 37923732

[262] Hagihara H. , Murano T. , Ohira K. , Miwa M. , Nakamura K. , and Miyakawa T. , Expression of Progenitor Cell/Immature Neuron Markers Does Not Present Definitive Evidence for Adult Neurogenesis, Molecular Brain. (2019) 12, no. 1, 10.1186/s13041-019-0522-8, 108. 31823803 PMC6902531

[263] Zhang L. , Xiong Z. , and Xiao M. , A Review of the Application of Spatial Transcriptomics in Neuroscience, Interdisciplinary Sciences: Computational Life Sciences. (2024) 16, no. 2, 243–260, 10.1007/s12539-024-00603-4. 38374297

[264] Simard S. , Rahimian R. , and Davoli M. A. , et al.Spatial Transcriptomic Analysis of Adult Hippocampal Neurogenesis in the Human Brain, Journal of Psychiatry and Neuroscience. (2024) 49, no. 5, E319–E333, 10.1503/jpn.240026. 39414359 PMC11495544

[265] Sa S. , Zhang H. , and Feng Y. , et al.Region-Specific Neurovascular Decoupling Associated With Cognitive Decline in Parkinson’s Disease, Frontiers in Aging Neuroscience. (2021) 13, 10.3389/fnagi.2021.770528, 770528. 34867297 PMC8636132

[266] Donato A. J. , Machin D. R. , and Lesniewski L. A. , Mechanisms of Dysfunction in the Aging Vasculature and Role in Age-Related Disease, Circulation Research. (2018) 123, no. 7, 825–848, 10.1161/CIRCRESAHA.118.312563. 30355078 PMC6207260

[267] Kohn J. C. , Lampi M. C. , and Reinhart-King C. A. , Age-Related Vascular Stiffening: Causes and Consequences, Frontiers in Genetics. (2015) 06, 10.3389/fgene.2015.00112, 112. PMC4396535 25926844

[268] Picca A. , Ferri E. , Calvani R. , Coelho-Júnior H. J. , Marzetti E. , and Arosio B. , Age-Associated Glia Remodeling and Mitochondrial Dysfunction in Neurodegeneration: Antioxidant Supplementation as a Possible Intervention, Nutrients. (2022) 14, no. 12, 10.3390/nu14122406, 2406. 35745134 PMC9230668

[269] Victorelli S. , Salmonowicz H. , and Chapman J. , et al.Apoptotic Stress Causes mtDNA Release During Senescence and Drives the SASP, Nature. (2023) 622, no. 7983, 627–636, 10.1038/s41586-023-06621-4. 37821702 PMC10584674

[270] Takasugi M. , Emerging Roles of Extracellular Vesicles in Cellular Senescence and Aging, Aging Cell. (2018) 17, no. 2, 10.1111/acel.12734, e12734. 29392820 PMC5847882

[271] Misawa T. , Tanaka Y. , Okada R. , and Takahashi A. , Biology of Extracellular Vesicles Secreted From Senescent Cells as Senescence-Associated Secretory Phenotype Factors, Geriatrics & Gerontology International. (2020) 20, no. 6, 539–546, 10.1111/ggi.13928. 32358923

[272] Mensà E. , Guescini M. , and Giuliani A. , et al.Small Extracellular Vesicles Deliver miR-21 and miR-217 as pro-Senescence Effectors to Endothelial Cells, Journal of Extracellular Vesicles. (2020) 9, no. 1, 10.1080/20013078.2020.1725285, 1725285. 32158519 PMC7048230

[273] Hernandez I. , Botana L. , and Diez-Mata J. , et al.CAP1: A Novel Extracellular Vesicle Marker Linked to Endothelial Senescence in Atherosclerosis, Biology Direct. (2025) 20, no. 1, 10.1186/s13062-025-00646-7, 46. 40189560 PMC11974053

[274] Tanaka Y. and Takahashi A. , Senescence-Associated Extracellular Vesicle Release Plays a Role in Senescence-Associated Secretory Phenotype (SASP) in Age-Associated Diseases, The Journal of Biochemistry. (2021) 169, no. 2, 147–153, 10.1093/jb/mvaa109. 33002139

[275] Han J. , Xu K. , Xu T. , Song Q. , Duan T. , and Yang J. , The Functional Regulation Between Extracellular Vesicles and the DNA Damage Responses, Mutation Research-Reviews in Mutation Research. (2025) 795, 10.1016/j.mrrev.2025.108532, 108532. 39828141

[276] Lochhead J. J. , Yang J. , Ronaldson P. T. , and Davis T. P. , Structure, Function, and Regulation of the Blood-Brain Barrier Tight Junction in Central Nervous System Disorders, Frontiers in Physiology. (2020) 11, 10.3389/fphys.2020.00914, 914. 32848858 PMC7424030

[277] Watters A. K. , Rom S. , and Hill J. D. , et al.Identification and Dynamic Regulation of Tight Junction Protein Expression in Human Neural Stem Cells, Stem Cells and Development. (2015) 24, no. 12, 1377–1389, 10.1089/scd.2014.0497. 25892136 PMC4485374

[278] Yin Y. , Chen H. , Wang Y. , Zhang L. , and Wang X. , Roles of Extracellular Vesicles in the Aging Microenvironment and Age-Related Diseases, Journal of Extracellular Vesicles. (2021) 10, no. 12, 10.1002/jev2.12154, e12154. 34609061 PMC8491204

[279] Manni G. , Buratta S. , and Pallotta M. T. , et al.Extracellular Vesicles in Aging: An Emerging Hallmark?, Cells. (2023) 12, no. 4, 10.3390/cells12040527, 527. 36831194 PMC9954704

[280] Sahu A. , Clemens Z. J. , and Shinde S. N. , et al.Regulation of Aged Skeletal Muscle Regeneration by Circulating Extracellular Vesicles, Nature Aging. (2021) 1, no. 12, 1148–1161, 10.1038/s43587-021-00143-2. 35665306 PMC9165723

[281] Sun Z. , Gao Z. , Wu J. , Zheng X. , Jing S. , and Wang W. , MSC-Derived Extracellular Vesicles Activate Mitophagy to Alleviate Renal Ischemia/Reperfusion Injury via the miR-223-3p/NLRP3 Axis, Stem Cells International. (2022) 2022, no. 1, 10.1155/2022/6852661, 6852661. 35646124 PMC9142309

[282] Rudraprasad D. , Nirmal J. , Mishra D. K. , and Joseph J. , RNA-Sequencing Reveals the Modulation of the NLRP3 Inflammasome by miR-223-3p in Extracellular Vesicles in Bacterial Endophthalmitis, Investigative Ophthalmology & Visual Science. (2025) 66, no. 4, 10.1167/iovs.66.4.53, 53. PMC12013669 40249602

[283] Chen X. , Luo Y. , and Zhu Q. , et al.Small Extracellular Vesicles From Young Plasma Reverse Age-Related Functional Declines by Improving Mitochondrial Energy Metabolism, Nature Aging. (2024) 4, no. 6, 814–838, 10.1038/s43587-024-00612-4. 38627524 PMC11186790

[284] Lei Z. , Krishnamachary B. , and Khan N. Z. , et al.Spinal Cord Injury Disrupts Plasma Extracellular Vesicles Cargoes Leading to Neuroinflammation in the Brain and Neurological Dysfunction in Aged Male Mice, Brain, Behavior, and Immunity. (2024) 120, 584–603, 10.1016/j.bbi.2024.07.005. 38986724 PMC11269008

[285] Booth L. N. and Brunet A. , The Aging Epigenome, Molecular Cell. (2016) 62, no. 5, 728–744, 10.1016/j.molcel.2016.05.013. 27259204 PMC4917370

[286] Audesse A. J. and Webb A. E. , Mechanisms of Enhanced Quiescence in Neural Stem Cell Aging, Mechanisms of Ageing and Development. (2020) 191, 10.1016/j.mad.2020.111323, 111323. 32781077 PMC7592125

[287] Feng X. , Jia M. , Cai M. , Zhu T. , Yang J.-J. , and Hashimoto K. , Central-Peripheral Neuroimmune Dynamics in Psychological Stress and Depression: Insights From Current Research, Molecular Psychiatry. (2025) 30, no. 10, 4881–4898, 10.1038/s41380-025-03085-y. 40610703 PMC12436188

[288] Feng X. , Zhao Y. , and Yang T. , et al.Glucocorticoid-Driven NLRP3 Inflammasome Activation in Hippocampal Microglia Mediates Chronic Stress-Induced Depressive-Like Behaviors, Frontiers in Molecular Neuroscience. (2019) 12, 10.3389/fnmol.2019.00210, 210. 31555091 PMC6727781

[289] Zhao M. , Wang W. , Jiang Z. , Zhu Z. , Liu D. , and Pan F. , Long-Term Effect of Post-traumatic Stress in Adolescence on Dendrite Development and H3K9me2/BDNF Expression in Male Rat Hippocampus and Prefrontal Cortex, Frontiers in Cell and Developmental Biology. (2020) 8, 10.3389/fcell.2020.00682, 682. 32850808 PMC7412801

[290] Garland E. L. , Hanley A. W. , Baker A. K. , and Howard M. O. , Biobehavioral Mechanisms of Mindfulness as a Treatment for Chronic Stress: An RDoC Perspective, Chronic Stress. (2017) 1, 10.1177/2470547017711912, 2470547017711912. 28840198 PMC5565157

[291] Bernabeu-Zornoza A. , Coronel R. , Palmer C. , López-Alonso V. , and Liste I. , Oligomeric and Fibrillar Species of Aβ42 Diversely Affect Human Neural Stem Cells, International Journal of Molecular Sciences. (2021) 22, no. 17, 10.3390/ijms22179537, 9537. 34502444 PMC8430695

[292] López-Toledano M. A. and Shelanski M. L. , Neurogenic Effect of β-aAmyloid Peptide in the Development of Neural Stem Cells, Journal of Neuroscience. (2004) 24, no. 23, 5439–5444, 10.1523/JNEUROSCI.0974-04.2004. 15190117 PMC6729298

[293] Demars M. , Hu Y. S. , Gadadhar A. , and Lazarov O. , Impaired Neurogenesis Is an Early Event in the Etiology of Familial Alzheimer’s Disease in Transgenic Mice, Journal of Neuroscience Research. (2010) 88, no. 10, 2103–2117, 10.1002/jnr.22387. 20209626 PMC3696038

[294] Meng J. X. , Zhang Y. , and Saman D. , et al.Hyperphosphorylated Tau Self-Assembles Into Amorphous Aggregates Eliciting TLR4-Dependent Responses, Nature Communications. (2022) 13, no. 1, 10.1038/s41467-022-30461-x, 2692. PMC9110413 35577786

[295] Chang E. H. , Adorjan I. , Mundim M. V. , Sun B. , Dizon M. L. , and Szele F. G. , Traumatic Brain Injury Activation of the Adult Subventricular Zone Neurogenic Niche, Frontiers in Neuroscience. (2016) 10, 10.3389/fnins.2016.00332, 332. 27531972 PMC4969304

[296] Tian Z. , Ji X. , and Liu J. , Neuroinflammation in Vascular Cognitive Impairment and Dementia: Current Evidence, Advances, and Prospects, International Journal of Molecular Sciences. (2022) 23, no. 11, 10.3390/ijms23116224, 6224. 35682903 PMC9181710

[297] Fitzgerald K. C. , Damian A. , Conway D. , and Mowry E. M. , Vascular Comorbidity Is Associated With Lower Brain Volumes and Lower Neuroperformance in a Large Multiple Sclerosis Cohort, Multiple Sclerosis Journal. (2021) 27, no. 12, 1914–1923, 10.1177/1352458520984746. 33416436 PMC8263795

[298] Kopalli S. R. , Behl T. , and Baldaniya L. , et al.Neuroadaptation in Neurodegenerative Diseases: Compensatory Mechanisms and Therapeutic Approaches, Progress in Neuro-Psychopharmacology and Biological Psychiatry. (2025) 139, 10.1016/j.pnpbp.2025.111375, 111375. 40280271

[299] Cuartero M. I. , García-Culebras A. , and Torres-López C. , et al.Post-stroke Neurogenesis: Friend or Foe?, Frontiers in Cell and Developmental Biology. (2021) 9, 10.3389/fcell.2021.657846, 657846. 33834025 PMC8021779

[300] Xu W. , Yu J.-T. , Tan M.-S. , and Tan L. , Cognitive Reserve and Alzheimer’s Disease, Molecular Neurobiology. (2015) 51, no. 1, 187–208, 10.1007/s12035-014-8720-y. 24794146

[301] Perneczky R. , Kempermann G. , and Korczyn A. D. , et al.Translational Research on Reserve Against Neurodegenerative Disease: Consensus Report of the International Conference on Cognitive Reserve in the Dementias and the Alzheimer’s Association Reserve, Resilience and Protective Factors Professional Interest Area working groups, BMC medicine. (2019) 17, no. 1, 47. 30808345 10.1186/s12916-019-1283-z PMC6391801

[302] Parent J. M. , Elliott R. C. , Pleasure S. J. , Barbaro N. M. , and Lowenstein D. H. , Aberrant Seizure-Induced Neurogenesis in Experimental Temporal Lobe Epilepsy, Annals of Neurology. (2006) 59, no. 1, 81–91, 10.1002/ana.20699. 16261566

[303] Koyama R. , Tao K. , and Sasaki T. , et al.GABAergic Excitation after Febrile Seizures Induces Ectopic Granule Cells and Adult Epilepsy, Nature Medicine. (2012) 18, no. 8, 1271–1278, 10.1038/nm.2850. 22797810

[304] Kasahara Y. , Nakashima H. , and Nakashima K. , Seizure-Induced Hilar Ectopic Granule Cells in the Adult Dentate Gyrus, Frontiers in Neuroscience. (2023) 17, 10.3389/fnins.2023.1150283, 1150283. 36937666 PMC10017466

[305] Althaus A. L. , Moore S. J. , Zhang H. , Du X. , Murphy G. G. , and Parent J. M. , Altered Synaptic Drive Onto Birthdated Dentate Granule Cells in Experimental Temporal Lobe Epilepsy, The Journal of Neuroscience. (2019) 39, no. 38, 7604–7614, 10.1523/JNEUROSCI.0654-18.2019. 31270158 PMC6750946

[306] Zhan R.-Z. , Timofeeva O. , and Nadler J. V. , High Ratio of Synaptic Excitation to Synaptic Inhibition in Hilar Ectopic Granule Cells of Pilocarpine-Treated Rats, Journal of Neurophysiology. (2010) 104, no. 6, 3293–3304, 10.1152/jn.00663.2010. 20881195 PMC3007662

[307] Danzer S. , Mossy Fiber Sprouting in the Epileptic Brain: Taking on the Lernaean Hydra, Epilepsy Currents. (2017) 17, no. 1, 50–51, 10.5698/1535-7511-17.1.50. 28331473 PMC5340559

[308] Pierce J. P. , Melton J. , Punsoni M. , McCloskey D. P. , and Scharfman H. E. , Mossy Fibers Are the Primary Source of Afferent Input to Ectopic Granule Cells that Are Born after Pilocarpine-Induced Seizures, Experimental Neurology. (2005) 196, no. 2, 316–331, 10.1016/j.expneurol.2005.08.007. 16342370 PMC1431686

[309] Abbate C. , The Adult Neurogenesis Theory of Alzheimer’s Disease, Journal of Alzheimer’s Disease. (2023) 93, no. 4, 1237–1276, 10.3233/JAD-221279. PMC10357199 37182879

[310] Richetin K. , Steullet P. , and Pachoud M. , et al.Tau Accumulation in Astrocytes of the Dentate Gyrus Induces Neuronal Dysfunction and Memory Deficits in Alzheimer’s Disease, Nature Neuroscience. (2020) 23, no. 12, 1567–1579, 10.1038/s41593-020-00728-x. 33169029

[311] Naganishi S. , Hagihara H. , and Miyakawa T. , Gene Expression Signatures of Immaturity, Decreased pH, and Neural Hyperexcitation in the Hippocampus of Alzheimer’s Disease Model Mice, Neuropsychopharmacology Reports. (2025) 45, no. 1, 10.1002/npr2.70001, e70001. 39907034 PMC11795175

[312] Brant B. , Stern T. , and Shekhidem H. A. , et al.IQSEC2 Mutation Associated With Epilepsy, Intellectual Disability, and Autism Results in Hyperexcitability of Patient-Derived Neurons and Deficient Synaptic Transmission, Molecular Psychiatry. (2021) 26, no. 12, 7498–7508, 10.1038/s41380-021-01281-0. 34535765 PMC8873005

[313] Velikic G. , Maric D. M. , and Maric D. L. , et al.Harnessing the Stem Cell Niche in Regenerative Medicine: Innovative Avenue to Combat Neurodegenerative Diseases, International Journal of Molecular Sciences. (2024) 25, no. 2, 10.3390/ijms25020993, 993. 38256066 PMC10816024

[314] Eftekhari B. S. , Eskandari M. , Janmey P. A. , Samadikuchaksaraei A. , and Gholipourmalekabadi M. , Surface Topography and Electrical Signaling: Single and Synergistic Effects on Neural Differentiation of Stem Cells, Advanced Functional Materials. (2020) 30, no. 25, 10.1002/adfm.201907792, 1907792.

[315] Akassoglou K. , Agalliu D. , and Chang C. J. , et al.Neurovascular and Immuno-Imaging: From Mechanisms to Therapies Proceedings of the Inaugural Symposium, Frontiers in Neuroscience. (2016) 10, 46. 26941593 10.3389/fnins.2016.00046 PMC4761864

[316] Weissman T. A. and Pan Y. A. , Brainbow: New Resources and Emerging Biological Applications for Multicolor Genetic Labeling and Analysis, Genetics. (2015) 199, no. 2, 293–306, 10.1534/genetics.114.172510. 25657347 PMC4317644

[317] Hampel H. , Nisticò R. , and Seyfried N. T. , et al.Omics Sciences for Systems Biology in Alzheimer’s Disease: State-of-the-Art of the Evidence, Ageing Research Reviews. (2021) 69, 10.1016/j.arr.2021.101346, 101346. 33915266

[318] Harrell C. R. , Volarevic A. , Djonov V. , and Volarevic V. , Mesenchymal Stem Cell-Derived Exosomes as New Remedy for the Treatment of Neurocognitive Disorders, International Journal of Molecular Sciences. (2021) 22, no. 3, 10.3390/ijms22031433, 1433. 33535376 PMC7867043

[319] Bach M. , Peeler M. , Caunca M. , Olusanya B. O. , Rosendale N. , and Gano D. , Brain Health Equity and the Influence of Social Determinants Across the Life Cycle, Seminars in Fetal and Neonatal Medicine. (2024) 29, 101553. 39537455 10.1016/j.siny.2024.101553

[320] Sun B. , Wang M. , Hoerder-Suabedissen A. , Xu C. , Packer A. M. , and Szele F. G. , Intravital Imaging of the Murine Subventricular Zone With Three Photon Microscopy, Cerebral Cortex. (2022) 32, no. 14, 3057–3067, 10.1093/cercor/bhab400. 35029646 PMC9290563

[321] Jacobsen J. H. W. , Parker L. M. , and Everest-Dass A. V. , et al.Novel Imaging Tools for Investigating the Role of Immune Signalling in the Brain, Brain, Behavior, and Immunity. (2016) 58, 40–47, 10.1016/j.bbi.2016.04.014. 27129634

[322] Booth T. C. , Williams M. , Luis A. , Cardoso J. , Ashkan K. , and Shuaib H. , Machine Learning and Glioma Imaging Biomarkers, Clinical Radiology. (2020) 75, no. 1, 20–32, 10.1016/j.crad.2019.07.001. 31371027 PMC6927796

[323] Bressan R. B. , Dewari P. S. , and Kalantzaki M. , et al.Efficient CRISPR/Cas9-Assisted Gene Targeting Enables Rapid and Precise Genetic Manipulation of Mammalian Neural Stem Cells, Development. (2017) 144, no. 4, 635–648, 10.1242/dev.140855. 28096221 PMC5312033

[324] Dulken B. W. , Leeman D. S. , Boutet S. C. , Hebestreit K. , and Brunet A. , Single-Cell Transcriptomic Analysis Defines Heterogeneity and Transcriptional Dynamics in the Adult Neural Stem Cell Lineage, Cell Reports. (2017) 18, no. 3, 777–790, 10.1016/j.celrep.2016.12.060. 28099854 PMC5269583

[325] Vandereyken K. , Sifrim A. , Thienpont B. , and Voet T. , Methods and Applications for Single-Cell and Spatial Multi-Omics, Nature Reviews Genetics. (2023) 24, no. 8, 494–515, 10.1038/s41576-023-00580-2. PMC9979144 36864178

[326] Keele G. R. , Zhang J.-G. , and Szpyt J. , et al.Global and Tissue-Specific Aging Effects on Murine Proteomes, Cell Reports. (2023) 42, no. 7, 10.1016/j.celrep.2023.112715, 112715. 37405913 PMC10588767

[327] Sathyan S. , Ayers E. , and Gao T. , et al.Plasma Proteomic Profile of Age, Health Span, and All-cause Mortality in Older Adults, Aging Cell. (2020) 19, no. 11, 10.1111/acel.13250, e13250. 33089916 PMC7681045

[328] Li B. , Sierra A. , and Deudero J. J. , et al.Multitype Bellman-Harris Branching Model Provides Biological Predictors of Early Stages of Adult Hippocampal Neurogenesis, BMC Systems Biology. (2017) 11, no. S5, 10.1186/s12918-017-0468-3, 90. 28984196 PMC5629620

[329] Guenoun D. , Blaise N. , and Sellam A. , et al.Microglial Depletion, a New Tool in Neuroinflammatory Disorders: Comparison of Pharmacological Inhibitors of the CSF-1R, Glia. (2025) 73, no. 4, 686–700, 10.1002/glia.24664. 39719687 PMC11845850

[330] Fu H. , Zhao Y. , Hu D. , Wang S. , Yu T. , and Zhang L. , Depletion of Microglia Exacerbates Injury and Impairs Function Recovery After Spinal Cord Injury in Mice, Cell Death & Disease. (2020) 11, no. 7, 10.1038/s41419-020-2733-4, 528. 32661227 PMC7359318

[331] Ogrodnik M. , Evans S. A. , and Fielder E. , et al.Whole-Body Senescent Cell Clearance Alleviates Age-Related Brain Inflammation and Cognitive Impairment in Mice, Aging Cell. (2021) 20, no. 2, 10.1111/acel.13296, e13296. 33470505 PMC7884042

[332] Liu K. , Fan D. , and Wu H.-P. , et al.Senescent Microglia Mediate Neuroinflammation-Induced Cognitive Dysfunction by Selective Elimination of Excitatory Synapses in the Hippocampal CA1, Aging Cell. (2025) 24, no. 9, 10.1111/acel.70167, e70167. 40624910 PMC12419858

[333] Xu T. , Gan L. , and Chen W. , et al.Bridging Immune-Neurovascular Crosstalk via the Immunomodulatory Microspheres for Promoting Neural Repair, Bioactive Materials. (2025) 44, 558–571, 10.1016/j.bioactmat.2024.10.031. 39584066 PMC11583666

[334] Rhea E. M. , Logsdon A. F. , Banks W. A. , and Erickson M. E. , Intranasal Delivery: Effects on the Neuroimmune Axes and Treatment of Neuroinflammation, Pharmaceutics. (2020) 12, no. 11, 10.3390/pharmaceutics12111120, 1120. 33233734 PMC7699866

[335] Parveen S. , Impact of Calorie Restriction and Intermittent Fasting on Periodontal Health, Periodontology 2000. (2021) 87, no. 1, 315–324, 10.1111/prd.12400. 34463980

[336] Abdanipour A. , Karami B. , Fridoni M. J. , Mohamadi M. , and Fakheri F. , Synergistic Effect of Lovastatin and Selegiline on the Differentiation of Bone Marrow Stromal Cells Into Neuron-Like Cells, Jentashapir Journal of Cellular and Molecular Biology. (2023) 14, no. 4, 10.5812/jjcmb-141092.

[337] Roberts W. S. , Price S. , Wu M. , and Parmar M. S. , Emerging Gene Therapies for Alzheimer’s and Parkinson’s Diseases: An Overview of Clinical Trials and Promising Candidates, Cureus. (2024) 16, no. 8, 10.7759/cureus.67037. PMC11405083 39286667

[338] Banja J. D. , Ethical Considerations in Stem Cell Research on Neurologic and Orthopedic Conditions, PM&R. (2015) 7, no. 4S, S66–S75, 10.1016/j.pmrj.2014.10.016. 25595666

[339] Lizarraga K. J. , Gyang T. , and Benson R. T. , et al.Seven Strategies to Integrate Equity Within Translational Research in Neurology, Annals of Neurology. (2024) 95, no. 3, 432–441, 10.1002/ana.26873. 38270253 PMC10922988

[340] Spangenberg E. , Severson P. , and Hohsfield L. , et al.Sustained Microglial Depletion With CSF1R Inhibitor Impairs Parenchymal Plaque Development in an Alzheimer’s Disease Model, Nature Communications. (2019) 10, no. 1, 10.1038/s41467-019-11674-z, 3758. PMC6704256 31434879

[341] Henry R. J. , Ritzel R. M. , and Barrett J. P. , et al.Microglial Depletion With CSF1R Inhibitor During Chronic Phase of Experimental Traumatic Brain Injury Reduces Neurodegeneration and Neurological Deficits, The Journal of Neuroscience. (2020) 40, no. 14, 2960–2974, 10.1523/JNEUROSCI.2402-19.2020. 32094203 PMC7117897

[342] Casali B. T. , MacPherson K. P. , Reed-Geaghan E. G. , and Landreth G. E. , Microglia Depletion Rapidly and Reversibly Alters Amyloid Pathology by Modification of Plaque Compaction and Morphologies, Neurobiology of Disease. (2020) 142, 10.1016/j.nbd.2020.104956, 104956. 32479996 PMC7526856

[343] Bellver-Landete V. , Bretheau F. , and Mailhot B. , et al.Microglia Are an Essential Component of the Neuroprotective Scar That Forms After Spinal Cord Injury, Nature Communications. (2019) 10, no. 1, 10.1038/s41467-019-08446-0, 2-s2.0-85060934561, 518. PMC6355913 30705270

[344] Planas A. M. , Role of Microglia in Stroke, Glia. (2024) 72, no. 6, 1016–1053, 10.1002/glia.24501. 38173414

[345] Jin W.-N. , Shi S. X.-Y. , and Li Z. , et al.Depletion of Microglia Exacerbates Postischemic Inflammation and Brain Injury, Journal of Cerebral Blood Flow & Metabolism. (2017) 37, no. 6, 2224–2236, 10.1177/0271678X17694185, 2-s2.0-85019689599. 28273719 PMC5444553

[346] Zhou Z.-L. , Xie H. , and Tian X.-B. , et al.Microglial Depletion Impairs Glial Scar Formation and Aggravates Inflammation Partly by Inhibiting STAT3 Phosphorylation in Astrocytes After Spinal Cord Injury, Neural Regeneration Research. (2023) 18, no. 6, 1325–1331, 10.4103/1673-5374.357912. 36453419 PMC9838173

[347] Csik B. , Nyúl-Tóth Á. , and Gulej R. , et al.Senescent Endothelial Cells in Cerebral Microcirculation Are Key Drivers of Age-Related Blood–Brain Barrier Disruption, Aging cell. (2025) 24, no. 7, 10.1111/acel.70048, e70048. 40167015 PMC12266767

[348] Riessland M. , Ximerakis M. , Jarjour A. A. , Zhang B. , and Orr M. E. , Therapeutic Targeting of Senescent Cells in the CNS, Nature Reviews Drug Discovery. (2024) 23, no. 11, 817–837, 10.1038/s41573-024-01033-z. 39349637 PMC11927922

[349] Real M. G. C. , Falcione S. R. , and Boghozian R. , et al.Endothelial Cell Senescence Effect on the Blood-Brain Barrier in Stroke and Cognitive Impairment, Neurology. (2024) 103, no. 11, 10.1212/WNL.0000000000210063, e210063. 39541552

[350] Iwao T. , Takata F. , and Matsumoto J. , et al.Senescence in Brain Pericytes Attenuates Blood-Brain Barrier Function In Vitro: A Comparison of Serially Passaged and Isolated Pericytes From Aged Rat Brains, Biochemical and Biophysical Research Communications. (2023) 645, 154–163, 10.1016/j.bbrc.2023.01.037. 36689812

[351] Nikolakopoulou A. M. , Montagne A. , and Kisler K. , et al.Pericyte Loss Leads to Circulatory Failure and Pleiotrophin Depletion Causing Neuron Loss, Nature Neuroscience. (2019) 22, no. 7, 1089–1098, 10.1038/s41593-019-0434-z, 2-s2.0-85067868288. 31235908 PMC6668719

